# Sequence comparison of prefrontal cortical brain transcriptome from a tame and an aggressive silver fox (*Vulpes vulpes*)

**DOI:** 10.1186/1471-2164-12-482

**Published:** 2011-10-03

**Authors:** Anna V Kukekova, Jennifer L Johnson, Clotilde Teiling, Lewyn Li, Irina N Oskina, Anastasiya V Kharlamova, Rimma G Gulevich, Ravee Padte, Michael M Dubreuil, Anastasiya V Vladimirova, Darya V Shepeleva, Svetlana G Shikhevich, Qi Sun, Lalit Ponnala, Svetlana V Temnykh, Lyudmila N Trut, Gregory M Acland

**Affiliations:** 1 Baker Institute for Animal Health, Cornell University, Ithaca, NY 14853, USA; 2 Roche Diagnostics, Indianapolis, IN 46256, USA; 3 454 Life Sciences, Branford, CT 06405, USA; 4 Institute of Cytology and Genetics of the Russian Academy of Sciences, Novosibirsk, 630090, Russia; 5 Computational Biology Service Unit, Biotechnology Center, Cornell University, Ithaca, NY 14853, USA

## Abstract

**Background:**

Two strains of the silver fox (*Vulpes vulpes*), with markedly different behavioral phenotypes, have been developed by long-term selection for behavior. Foxes from the tame strain exhibit friendly behavior towards humans, paralleling the sociability of canine puppies, whereas foxes from the aggressive strain are defensive and exhibit aggression to humans. To understand the genetic differences underlying these behavioral phenotypes fox-specific genomic resources are needed.

**Results:**

cDNA from mRNA from pre-frontal cortex of a tame and an aggressive fox was sequenced using the Roche 454 FLX Titanium platform (> 2.5 million reads & 0.9 Gbase of tame fox sequence; >3.3 million reads & 1.2 Gbase of aggressive fox sequence). Over 80% of the fox reads were assembled into contigs. Mapping fox reads against the fox transcriptome assembly and the dog genome identified over 30,000 high confidence fox-specific SNPs. Fox transcripts for approximately 14,000 genes were identified using SwissProt and the dog RefSeq databases. An at least 2-fold expression difference between the two samples (p < 0.05) was observed for 335 genes, fewer than 3% of the total number of genes identified in the fox transcriptome.

**Conclusions:**

Transcriptome sequencing significantly expanded genomic resources available for the fox, a species without a sequenced genome. In a very cost efficient manner this yielded a large number of fox-specific SNP markers for genetic studies and provided significant insights into the gene expression profile of the fox pre-frontal cortex; expression differences between the two fox samples; and a catalogue of potentially important gene-specific sequence variants. This result demonstrates the utility of this approach for developing genomic resources in species with limited genomic information.

## Background

Until recently, genome wide sequencing has been cost limited to relatively few species. Newer sequencing technologies, offering rapid high throughput sequencing, reduction of sequencing cost, and complementary software have changed the situation dramatically, making these methods more readily considered as standard molecular tools for biology and medicine [1-9]. Transcriptome sequencing in particular can now be undertaken by individual research groups to generate genomic information for novel or "orphan" species of scientific interest [10-14]. One such species is the red fox (*Vulpes vulpes*), including its farm bred coat color variant, the silver fox.

The red fox is one of the most widely distributed mammalian species across the globe [15,16] - inhabiting the Northern Hemisphere naturally and Australia by introduction. Foxes live in a wide range of temperature zones, geographical regions, wild and urban environments [16,17] and are close relatives of the domestic dog (*Canis lupus familiaris*). The grey wolf (*Canis lupus lupus*) and the fox diverged from a common ancestor about 10 million years ago [18], and the dog was domesticated from the grey wolf at least 15,000 years ago [19-24]. In contrast, the red fox -- or, more precisely, the silver fox -- has only recently been domesticated as part of a deliberate scientific experiment at the Institute of Cytology and Genetics (ICG) of the Russian Academy of Sciences in the second part of the twentieth century [for review [25-28]].

Starting from a farm-bred population of silver foxes, the ICG selectively bred foxes for tameness for over 50 generations [25-27]. Foxes from this tame strain exhibit human-directed behavior resembling that of domestic dogs http://cbsu.tc.cornell.edu/ccgr/behaviour/Index.htm[29]. In parallel, the ICG also undertook a selective breeding program to develop another strain of foxes that are aggressive and difficult to handle. Both strains of foxes are maintained at the ICG as outbred populations. The genetic basis of these tame and aggressive behavioral phenotypes was confirmed in experiments that included cross-fostering of pups, transplantation of embryos and cross-breeding experiments [30,25,26,32]. These fox strains present a robust model, intermediate between rodent and primate in biological complexity, for the study of genetics of affiliative versus aggressive behaviors.

By exploiting genomic resources developed for the domestic dog - the only canid species with a sequenced genome, to date - we recently began molecular genetic studies of these selectively bred tame and aggressive fox strains. To this end, we developed crossbred F1, back-cross and intercross pedigrees segregating behavioral differences derived from the founder strains; a meiotic linkage map of the fox genome based on 385 canine-derived microsatellite markers [33,34,32]; and, using these resources, mapped several quantitative trait loci (QTL) in the fox genome associated with behavior [32].

Although genetic mapping with canine derived microsatellite markers allowed identification of several behavioral loci in fox genome, this type of genetic analysis has inherent limitations in its mapping power. Single nucleotide polymorphism (SNP) markers would be much more powerful for fine mapping these behavioral loci, and identification of haplotypes cosegregating with QTLs that were under selection in the founder strains. Unfortunately, the majority of dog SNPs are not informative in foxes and fox-specific SNPs need to be identified to move this study forward.

To this end we undertook deep transcriptome sequencing of pre-frontal brain samples from one tame and one aggressive fox. Because pre-frontal cortex is a brain area relatively easy to identify, samples from different individuals can be readily collected in a relatively consistent manner. Furthermore, the role of this brain region in aspects of behavior is well established [35], and thus particularly relevant to the study. Comparison of gene expression profiles between a tame and an aggressive individual offers insights into functional differences in the pre-frontal brain cortex. Comparison of transcriptome sequences of the same genes between the tame and aggressive fox samples, has identified a large set of informative SNP markers and begun a catalogue of gene-specific sequence variants between the two strains.

## Results

### 454 GS FLX Titanium sequencing the transcriptome of the silver fox prefrontal cortex

**Figure 1:**
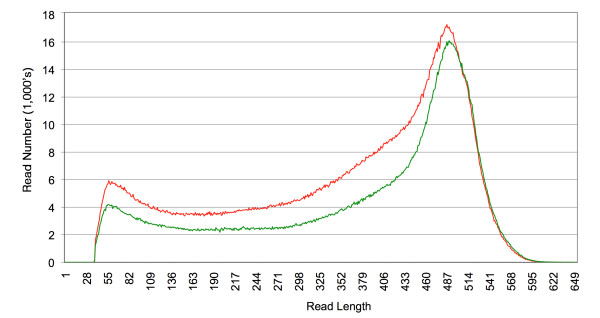
**Comparison of read length distributions for transcriptome sequences of one tame and one agressive fox**. Reads were generated from cDNA from prefrontal cortex using the Roche 454 FLX Titanium platform. Data represents a total of 6 runs comprising 5 half full runs for one tame individual (green) and 6 half full runs for one aggressive fox (red). Reads were quality filtered with standard parameters and cleaned of adaptor sequences. For statistics of reads see table 1.

Sequencing of non-normalized cDNA libraries from prefrontal cortex of one tame and one aggressive male fox after image analysis, signal processing and data filtering yielded 2,565,892 reads (0.96 Gb) from the tame fox sample and 3,379,343 reads (1.21 Gb) from the aggressive fox sample (Table 1). Distribution of filtered read lengths in each sample is presented in Figure 1. From the tame sample, the average read length was 376 bp (SD = 148 bp), and the median 433 bp. From the aggressive, the average was 358 bp (SD = 146 bp), and the median 403 bp. The primary sequencing data have been deposited in the GeneBank Sequence Read Archive (SRA) under accession number (SRA029285.1).

**Table 1:** Silver fox transcriptome: summary statistics of reads and assemblies.

Run	Total number of reads	Number of filtered bases (Gb)	**Average (median) read length in bp +/- S.D**.	Fully aligned (%)*	Partially aligned (%)*	Number of Isogroups*	Number of Isotigs*	Average Isotig Length	N50 Isotig Length	Number of Contigs* (length >= 100 bp)	Total number of bases in contigs at least 100 bp in length	Singletons* (%)	Repeats* (%)*	Outliers* (%)*	Too Short* (%)*
**Tame**	2,565,892	0.96	376 +/-148 (433)	1,921,335 (74.9%)	250,138 (9.7%)	37,679	50,311	1,565	2,473	55,656	52,072,035	287,577 (11.2%)	1,321 (0.1%)	78,309 (3.1%)	26,993 (1.1%)

**Aggressive**	3,379,343	1.21	358 +/-146 (403)	2,458,444 (72.7%)	338,660 (10.0%)	44,791	60,492	1,593	2,647	67,078	60,587,572	426,108 (12.6%)	1,023 (0.1%)	118,205 (3.6%)	36,637 (1.1%)

**All Together**	5,945,235	2.17		4,551,142 (76.6%)	571,726 (9.6%)	59,713	87,400	1,820	3,293	96,461	83,773,326	562,591 (9.5%)	14,192 (0.2%)	181,469 (3.1%)	63,630 1.1%

The number of reads is tabulated for both samples (Tame, Aggressive), both individually and all together, with the total number of bases for each set that passed filtering criteria and were used for mapping against the canine RefSeq database, together with analytic results of the corresponding fox transcriptome assemblies. **Isogroups: **an isogroup is a collection of contigs containing reads with implied connections between them, analogous to individual genes; **Isotigs: **an isotig is meant to be analogous to an individual transcript or splice variant; **Contigs: **a contig represents the consensus sequence of a self-consistent set of overlapping reads, roughly analogous to exons; **Singletons: **reads that did not overlap with any other reads in the input; **Repeats: **reads that were either inferred to be repetitive early in the assembly process or determined to partially overlap a contig; **Outliers**: reads that were identified by the GS *De Novo *Assembler as problematic, and were excluded from the final contigs; **Too Short**: the trimmed read was too short (< 50bp) to used in the computation. (see also Methods).

### Assembly of fox transcriptome sequencing reads

Fox sequencing reads that passed quality filtering were assembled *de novo *in three different manners: i) all reads together; ii) reads from the tame sample alone; iii) reads from the aggressive sample alone (see Table 1). Over 70% of reads were fully assembled, and close to a further 10% of reads were partly assembled in both the assembly of all reads together and in each individual assembly run (Table 1).

### BLAT of fox contigs to the dog genome

Among all contigs identified in the assembly of all reads together we selected the 96,461 contigs that were at least 100 bp long (Table 1). BLAT of these contigs against the canine genome sequence assembly (CanFam2) successfully located 91,262 contigs. The output file of BLAT results was then visualized on the UCSC Genome browser (Additional file 1, Figure S1). Alignment of fox contigs against the dog genome allowed quick visual examination of the genes represented in the fox transcriptome, revealed gene structure and possible splice variants.

### BLASTX of fox isotigs against the SwissProt data base

To estimate the number of genes well represented in this fox transcriptome, all isotigs identified in the assembly of all fox reads together (Table 1) were aligned against the SwissProt database using BLASTX. Of these, 46,776 isotigs (53.52% of the total number of isotigs) yielded a hit to one or more proteins in the database. Only the best hit for each isotig was selected. Successfully aligned isotigs belonged to 21,307 isogroups (35.38% of the total number of isogroups).

In total, 13,624 genes were identified, of which 6,952 (51.03%) were in more than one isogroup. The total number of genes identified in the fox transcriptome was significantly lower than the number of isogroups identified in the assembly of fox reads.

### Putative homologs of canine genes in the fox transcriptome

Because a fox reference genome sequence assembly does not exist, and the dog is the fox's closest relative with a sequenced genome, the canine RefSeq database was used to map fox reads to specific genes by nucleotide sequence alignment. All (5,945,235) fox reads that passed filtering for quality control (Table 1), from the six sequencing runs, were mapped against the canine RefSeq database (see Methods and Table 2). In total, 23.55% of these reads mapped either fully (21.23%) or partially (2.32%) to a known gene in the canine RefSeq database (Table 2), and a total of 14,418 canine genes (72.6% of all genes in the canine RefSeq database) were mapped as homologs of at least one fox read (Additional file 2, Table S1). After excluding from the remaining reads those that were short, chimeric or mapped repetitively, 56.11% of the total number of reads did not map to the canine RefSeq database (Table 2).

**Table 2:** Summary statistics from mapping fox sequencing reads against the Canine RefSeq database.

Total number of reads	Fully mapped	Partially mapped	Unmapped	Repeats	Chimeric	Too short to map
5,945,235	21.23%	2.32%	56.11%	17.91%	1.38%	1.04%

### Completeness of the fox transcriptome

**Figure 2:**
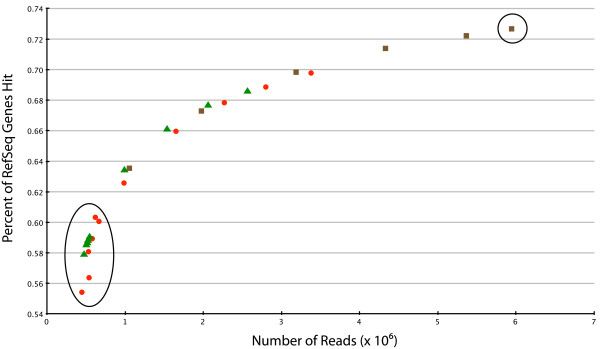
**Mapping fox sequencing reads against the canine RefSeq database: gene yield vs sequence depth**. The yield of identifiable genes from fox transcriptome sequencing is plotted as a function of sequencing depth. Sets of reads, each set representing half a sequencing run (approximately 500,000 reads) were combined variously into 9 datasets each representing from half a run (9 datapoints, circled cluster at left extremity of plot) to 11 half-runs (1 datapoint, circled, rightmost extremity of plot). The vertical axis gives the percentage of genes in the canine RefSeq database hit by at last one read for each datapoint. Green diamonds = datapoints comprised of tame reads only; red squares = aggressive reads only; brown circles = datapoint combines tame and aggressive reads.

Because non-normalized cDNA libraries were sequenced, it was expected that the most abundant transcripts in the fox transcriptome would be represented by a higher number of sequencing reads than would rarer transcripts. To estimate whether fox cDNA libraries were sequenced to a sufficient depth we mapped i) reads from individual 454 FLX sequencing runs (~500,000 reads per run) and ii) reads from several sequencing runs combined together, to make datasets of several different sizes, against the dog RefSeq database in separate experiments (Figure 2). Mapping all available reads from the tame (2.5 sequencing runs) and aggressive (3 sequencing runs) samples in separate experiments identified 13,618 and 13,855 of dog gene orthologs, respectively. The number of genes identified in the canine RefSeq database by mapping 4, 5, and 5.5 sequencing runs (the three data points furthest right on the plot) was very similar in each case (14,175; 14,339; and 14,429 genes, respectively), suggesting that the data plot (Figure 2) was approaching its saturation point. The plot likewise showed that increasing the depth of sequencing data from 5 to 10 half full runs identified only about 5% more genes.

**Figure 3:**
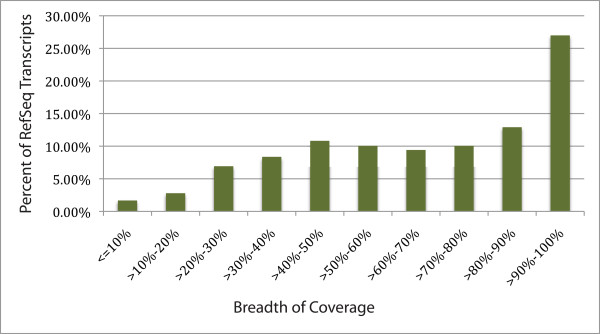
**Mapping fox sequencing reads against the canine RefSeq database: breadth of coverage**. For each accession number in the canine RefSeq database to which at least one fox read mapped, the breadth of coverage was calculated as the percent length of the canine transcript covered by all fox reads mapping to that accession number. The breadth of coverage appears to be bivariate.

The average breadth of coverage of dog RefSeq transcripts that were hit by at least one fox read was 66.4% (Figure 3). The average depth of coverage for dog RefSeq sequences that had at least one hit was 3.5 for tame and 4.0 for aggressive. In determining breadth of coverage, we used a conservative approach and counted only reads which were uniquely mapped to a single transcript by the 454 GS Reference Mapper. Because the reads corresponding to multiple splice variants were counted only for a single splice variant (or not counted at all), it is likely that the average depth of coverage of dog transcripts was underestimated. The fact that the breadth of coverage of dog transcripts mapped by reads from two individual samples or both samples was almost identical suggests that an average breadth of coverage at ~66% is the practical maximum we can expect to obtain using our mapping approach.

### Identification of fox SNPs

Sequence differences between dog and fox and among fox chromosomes were identified by mapping fox reads against the canine genome sequence assembly, and contigs from the assembly of all fox reads together.

#### •SNPs identified by mapping fox sequencing reads against the dog genome

To identify nucleotide differences between the dog and fox genomes and between the two foxes sequenced, we mapped all fox reads against the repeat-masked dog genomic sequence (CanFam2) (Table 3). In total, 35.09% of reads mapped fully (29.97%) or partially (5.12%) to the dog genome; 28.36% of fox reads mapped chimerically, with non-overlapping parts of the read aligned to distant locations in the dog genome. Reads that mapped to multiple locations (repeats, 3.13%) and reads that were too short to map reliably (1.04%) were excluded from further analysis (Table 3). The mapping run created a total of 170,099 dog-fox contigs containing 115,987,304 bases.

**Table 3:** Summary statistics from mapping fox sequencing reads against the repeatmasked CanFam2 sequence assembly of the dog genome.

Total number of reads	Fully mapped	Partially mapped	Unmapped	Repeats	Chimeric	Too short to map
5,945,235	29.97%	5.12%	32.38%	3.13%	28.36%	1.04%

Comparison of sequences from the four fox chromosomes represented in the present study with the one canine reference chromosome (i.e. CanFam2) identified a total of 991,041 sequence differences, including single nucleotide differences, indels and 2-3 nucleotide differences. For 3,511 of these differences only 1-5% of fox reads were in disagreement with the dog sequence. These latter differences were deemed less likely to be true nucleotide differences and were excluded from further analysis. This left 987,530 differences representing one difference (either between dog and fox or among fox chromosomes) for every 117 bases of dog-fox contigs. Of these 987,530 differences, 898,940 were fox-versus-dog differences, where at least 95% of fox reads were in agreement with each other, and differed from the dog sequence. This represented one difference per 129 mapped bases. An example of one such dog-fox nucleotide difference is presented in Supplementary Figure 2a (Additional file 3). In total, 88,590 differences (1 per 1,309 mapped bases) were identified among fox reads that aligned to the dog genome, an example of which is presented in Supplementary Figure 2b (Additional file 3).

The set of differences where the fox reads differed from each other were filtered to obtain 19,245 high confidence SNPs.

#### • SNPs identified by mapping fox reads against the assembled fox transcriptome sequence

A total of 96,461 fox contigs that were at least 100 bp long, from the assembly of all reads together (Table 1), were used as a reference for mapping fox reads. In total, 85,064 differences between fox reads were identified. These polymorphisms included SNPs, indels, and 2-3 nucleotide polymorphisms. After applying filtering criteria (see Methods section) 17,328 high-confidence fox SNPs were identified. Contigs containing high-confidence SNPs were selected and localized in the dog genome by BLAT, and the output file was visualized on the UCSC Genomic Browser.

#### • Non-redundant set of high-confidence fox-specific SNPs

High confidence fox SNPs were selected using parameters described in the Methods section. SNPs identified by both methods (mapping fox reads against the dog genome and against the fox transcriptome assembly) were checked for redundancy and duplicates were deleted. The non-redundant set contained 30,491 SNPs. SNPs were classified into four categories according to the number of alleles observed in each sample (Table 4).

**Table 4:** Numbers of SNPs detected, and their informativeness, in tame and aggressive fox samples.

	Homozygous Tame	Heterozygous Tame
Homozygous Aggressive	7,260	7,315

Heterozygous Aggressive	11,530	4,386

SNPs were classified by the observed genotypes in the tame and aggressive individuals, as either homozygous in both (i.e. for opposite alleles), heterozygous in both, or homozygous in one individual and heterozygous in the other.

**Figure 4:**
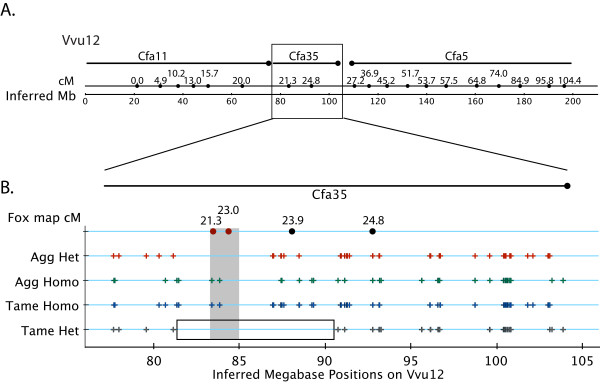
**Portion of fox chromosome 12 (VVU12) demonstrating SNP distribution and informativeness**. The region of VVU12 that is homogous to canine chromosome 35 is graphed to indicate the position and zygosity of SNPs detected by transcriptome analysis of one tame and one aggressive fox. The top row/lines indicates the alignment of CFA35 with this part of VVU12; the next row (Fox map cM) provides fox meiotic map distances; and the bottom row indicates estimated position in megabases, based on extrapolation from the canine genome sequence assembly. On the four central tracks, each SNP is represented by a single tick mark (+) for each individual (Aggressive or Tame), on either the heterozygous or homozygous track for that individual. Similar data is presented for all fox autosomes in Supplementary Figure 3. The grey box highlights a region that has previously been shown [32] to harbor a quantitative trait locus (QTL) associated with specific components of fox behavior with an association peak at 23 cM. The aggressive and tame individuals are homozygous for opposite SNP alleles in this interval. The outlined box on the Tame Het row indicates how the region of homozygosity is extended in the tame individual.

In total, 29,834 of these high confidence fox SNPs were localized in the dog genome and the inferred location of 29,368 of these SNPs on fox chromosomes was assigned using the comparative fox/dog map (Additional file 4, Figure S3). The informativeness of each SNP in each sample is also represented on fox chromosome graphs (Additional file 4, Figure S3). Figure 4 shows the distribution and zygosity of SNPs in a segment of VVU12 that has conserved synteny to dog chromosome 35. This region corresponds to part of a QTL interval identified by genetic mapping of behavioural phenotypes in fox experimental pedigrees [32]. Note that the aggressive and tame individuals are homozygous for opposite SNP alleles in this interval. In the tame animal, the region of homozygosity extends even further.

### SNP validation

To establish parameters for filtering high-confidence fox SNPs (see Methods section) we selected a subset of SNPs for validation by Sanger sequencing. Three sets of SNPs were selected. The first set included 20 SNPs in which the minor allele was present in at least 25% of the sequencing reads per sample and each allele was present in more than three reads in one individual (Additional file 5, Table S2a); the second set included 17 SNPs in which the minor allele was present in at least 25% of sequencing reads per sample and by exactly three reads in one individual (Additional file 5, Table S2b); the third set included 15 SNPs in which the minor allele was present in at least three reads between the two individuals (Additional file 5, Table S2c). SNPs that mapped to the canine X chromosome and showed heterozygosity in at least one sample were filtered out (except for SNPs that mapped to the pseudoautosomal region of CFAX) and not included in the SNP validation study. Of 51 SNPs, all but one (SNP #3-15) were validated by Sanger sequencing (Additional file 5, Table S2). Allele heterozygosity, identified by Sanger and 454 sequencing for each SNP in each individual, differed for two SNPs in the first dataset (Additional file 5, Table S2a), five SNPs in the second dataset (Additional file 5, Table S2b), and two SNPs in the third data set (Additional file 5, Table S2c). For all these SNPs except SNP #3-6 one of the two individuals was homozygous by 454 sequencing, but heterozygous by Sanger sequencing (Additional file 5 Table S2). The allele identified by Sanger sequencing corresponded to the second allele observed in another individual. The opposite situation was observed for SNP #3-6, with two SNP alleles identified in the tame individual by 454 sequencing but only one by Sanger sequencing (Additional file 5, Table S2c).

All SNPs in the first two data sets were validated by Sanger sequencing, indicating that the selection parameters applied identified high-confidence SNPs reliably. Over 90% of SNPs in the third data set (selected with modestly lower stringency filtering parameters) were also validated. Sanger resequencing of SNPs did find, however, that a second SNP allele was not always identified in the 454 sequencing datasets at the coverage cutoff used for selection of high-confidence SNPs (Additional file 5, Table S2). Indeed, all the minor SNP alleles not identified by 454 sequencing were among those with the lowest read coverage depth (~3-5× per sample, see Additional file 5, Table S2).

### Distribution of fox SNPs among different types of genomic regions

BLAT of SNP-bearing fox amplicons to the dog genome localized a subset of SNPs to non-coding regions of the dog genome. To evaluate the distribution of fox SNPs among known coding and non-coding regions we selected 100 random SNPs that passed defined stringent filtering parameters (see method section) and localized these SNPs in the dog genome (CanFam2) using the UCSC genome browser. In total, 77 SNPs were identified in exons or UTRs, 17 in introns or non-coding regions, and 6 SNPs were not localized unambiguously in the dog genome (Table 5). These results demonstrate that although the majority of high-confidence SNPs identified in the fox transcriptome correspond to translated sequences, our reads also contain sequences from non-coding genomic regions or regions not currently known to be transcribed in the dog genome.

**Table 5:** Mapping of SNPs, identified from the fox transcriptome, onto the dog genome sequence.

Number of SNPs	Type of genomic region
37	coding

9	intron

40	utr

8	non-gene

5	repetitive

1	unknown

100 randomly selected high-confidence fox SNPs were mapped onto the dog genome sequence assembly (CanFam2) to identify their distribution among different types of genome sequence.

### Comparison of gene expression between the two fox samples

**Figure 5:**
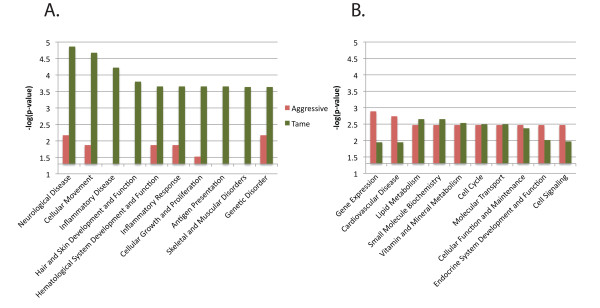
**Biofunction groups of genes expressed differentially between tame and aggressive foxes**. Expression differences between the tame and aggressive individuals were evaluated as the proportionate read count per gene. Genes with at least a 2-fold difference in expression (at p < 0.05) were sorted into Biofunction groups by Ingenuity IPA (Version 8.6). Biofunction groups were then ranked by the negative logarithm of the biofunction p-value, which estimates the probability that the biofunction group is over-represented in the set of differentially expressed genes. The vertical axis gives the statistical support for each bioinformatic group being upregulated in the tame (green) or aggressive individual (red) (a). The 10 highest ranked biofunction groups representing genes with higher expression in the tame individual. (b). The 10 highest ranked biofunction groups representing genes with higher expression in the aggressive individual.

A total of 27 genes had a tenfold difference of expression between the tame and aggressive individuals with p < 0.05. 335 genes had at least a twofold difference of expression and p < 0.05 (280 up in tame, 55 up in aggressive). Genes with differences in expression between the two individuals were classified into biofunctional groups, using Ingenuity IPA http://www.ingenuity.com[36]. The 10 most statistically significant biofunction groups for both the tame and the aggressive upregulated genes are shown in Figure 5. Networks were also analyzed, and a network of 16 genes was found in the set of upregulated genes from the aggressive individual that contains genes involved in the Behavior, Nervous System Development and Function and Cardiovascular Development and Function biofunction groups (data not shown).

### Validation of gene expression by RT-qPCR

Eleven genes that showed expression differences between the two fox samples, in analysis of transcriptome data, were selected for validation by RT-qPCR. Genes were chosen that had more than one exon and did not belong to a highly conserved gene family. Differences in expression of selected genes varied between two samples in terms of number of reads, fold differences, and statistical significance of observed expression differences. Six genes in this set had higher expression levels (more sequencing reads) in the transcriptome of the aggressive fox, versus five in the tame individual. Primers were designed to successfully amplify unique products for nine of these genes, but not successfully for *PKHD1L1 *and *BTNL9 *- the latter two genes were thus excluded from further analysis.

The relative expression level was estimated for these nine genes (Table 6) by calculating the difference in expression between "tame" and "aggressive" samples. Three genes (*KCNMA1*, *ITG8A*, and *SCGN*) showed less than a two-fold difference between RT-qPCR results and transcriptome analysis. Five genes (*HTR2C*, *LCOR*, *CDON*, *PRRG2*, *LRRC20*) showed more than a two-fold lower expression difference in RT-qPCR experiments than in the transcriptome analysis, although the difference between the "tame" and "aggressive" samples was in the same direction as in the transcriptome analysis. For four of these genes the difference in expression between the "tame" and "aggressive" samples detected by RT-qPCR was less than two-fold. One gene, *SLITRK6*, showed a difference of expression by RT-qPCR in the opposite direction from that by transcriptome analysis (Table 6). Expression difference of *SLITRK6 *in the transcriptome analysis had less significant support (Z = 2.04; p > 0.04) among all genes analysed in RT-qPCR experiments.

**Table 6:** RT-qPCR validation of expression differences identified by transcriptome sequence analysis.

			Transcriptome Sequencing				Relative RT-qPCR		
**Accession Number**	**Gene**	**Fold difference between two samples**	**Z statistics**	**p value**	**Number of reads in the sample of tame fox**	**Number of reads in the sample of aggressive fox**	**Sample ratio**	**Fold difference between two samples**	**Sample ratio**

XM_848547	SCGN	2.24	6.28	3.28E-10	136	402	A>T	2.44	A>T

XM_544247	ITGA8	4.48	4.85	1.24E-06	68	20	T>A	5.55	T>A

XM_546414	CDON	5.95	3.51	4.44E-04	6	47	A>T	1.09	A>T

NM_001006648	HTR2C	9.22	2.69	0.01	14	2	T>A	2.89	T>A

NM_001003300	KCNMA1	2.19	2.65	0.01	26	75	A>T	1.43	A>T

XM_543946	LCOR	10.63	2.14	0.03	1	14	A>T	1.75	A>T

XM_849641	PRRG2	11.71	2.11	0.03	8	0	T>A	1.31	T>A

XM_846863	LRRC20	10.54	2.08	0.04	8	1	T>A	1.12	T>A

XM_542630	SLITRK6	9.87	2.04	0.04	1	13	A>T	***1.54***	***T>A***

Nine genes, identified as differentially expressed between the tame and aggressive fox by transcriptome sequence analysis, were selected for expression evaluation by relative RT-qPCR. Results are sorted by Z test p-value. Except for one gene (*SLITRK6*, result in bold and italic), which had the least significant Z test p-value, RT-qPCR consistently confirmed a difference of expression between the tame and aggressive samples in the same direction as had transcriptome analysis (compare columns 8 and 10).

## Discussion

This study was designed to expand genomic resources for the red (silver) fox, an emerging animal model for genetic research whose genome has not yet been sequenced. Using the 454 GS FLX Titanium platform we sequenced non-normalized cDNA libraries from brain samples of two farm-bred foxes with markedly different genetically determined behavioral phenotypes.

Samples for sequencing were selected from prefrontal cortex because this region is easily recognisable in the brain; can thus be collected in a consistent manner; and is related to behavior. The role of different subdivisions of prefrontal cortex in regulating various aspects of cognition, memory, emotions, and social behavior is reviewed in Clark *et al*., 2010 [35]. In humans, a link between brain damage of prefrontal cortex and aggressive behavior has been reported [37].

A total of 0.96 Gb of transcriptome sequencing data for the tame fox and 1.21 Gb for the aggressive fox were obtained. The depth of sequencing chosen for this study was based on previous experience sequencing non-normalized cDNA libraries [38]. The fox reads were assembled in three different combinations: i) only reads from the tame fox, ii) only reads from the aggressive fox; iii) all reads together. The percent of reads assigned into contigs in each of the three assemblies was very similar (Table 1).

The assembly software used, the 454 GS *De Novo *assembler ("Newbler"), takes into account the existence of gene splice variants. Newbler first assembles sets of self-consistent reads into contigs, which are the rough equivalent of exons. It then builds isotigs (the rough equivalent of transcripts) from contigs that are consistently connected by subpopulations of reads. When there are splice variants, contigs can be connected via different paths that are supported by read data, leading to multiple isotigs being generated. Isotigs are grouped into isogroups, which are the rough equivalent of genes. In the context of a transcriptome assembly by Newbler, contigs are the sections of isogroups where corresponding isotig sequences are the same. Consequently, the assembly produces two sets of sequences: a theoretically non-redundant set of contigs, and a set of isotigs which are candidate long- or full-length transcripts from multiple splice variants of genes, and contains some partially redundant sequence segments. Newbler is currently the only transcripome assembler that attempts this task, and is one of the mostly widely used assemblers for *de novo *assembly of organisms with few genomic recourses [39].

Comparison of transcriptome assemblies from different studies is inherently problematic because different assembly algorithms handle the complexities of the transciptome in a variety of ways. CAP3, which was used in the assembly of transcriptomes of two mammalian species -- the bank vole (*Myodes glareolus*) and the domestic ferret (*Mustela putorious furo*) [14,10] -- does not split reads into multiple contigs as Newbler does [39]. This makes the number of contigs non-comparable. One measure of comparison is the span of contigs, the total number of bases in the all contigs in an assembly. This number is available for the bank vole, as the number of contigs reported, 63,581, multiplied by the average length of the contigs, 418, for an assembly span of 30.6 Mb. Assembly of the fox transcriptome spans 84.1 Mb. Relative to the bank vole, the fox transcriptome was sequenced to approximately 6.2 times greater depth and the assembly span was higher by 2.7 times.

The number of isogroups, isotigs, and contigs identified in the assembly of all fox reads together was significantly higher than the number of isogroups, isotigs, and contigs identified in the separate assemblies of tame and aggressive reads (Table 1). Singleton reads in the individual assemblies, when assembled together, created new contigs, isotigs and isogroups. Furthermore, sequence variations between individuals increased the number of contigs and isotigs in isogroups, when these variations were sufficiently large for the assembly program to assume them to be different exons, or real differences in splice variants of some genes between the two individuals. The average isotig length and N50 isotig length were also longer in the assembly of all reads together, as would be expected from an increase in the amount of sequencing data (Table 1).

Because the fox genome has not yet been sequenced and a fox RefSeq database is thus not available, we used SwissProt and the dog RefSeq database to identify gene orthologs in the fox transcriptome. BLASTX of fox isotigs from the assembly of all reads together against SwissProt database and mapping of all fox reads against dog RefSeq database identified very similar number of genes (13,624 and 14,418 genes, respectively).

The number of identified genes in the fox transcriptome was significantly lower than the number of isogroups (59,713) found in the assembly of fox reads. The total number of isogroups was also larger than the total number of genes in mammalian genome. One hypothesis is that the amount of sequencing data used in the assembly was not sufficient to cover the full lengths of all fox gene transcripts, therefore some poorly covered genes might have been partitioned into two or more isogroups. Consistent with this hypothesis, BLASTX of fox isotigs against SwissProt identified 13,624 genes, of which 6,952 (51.03%) were found in more than one isogroup (see Results section). In addition, a subset of fox reads (Table 5) was mapped to non-gene regions in the dog genome. These regions are expected not to be present in the dog RefSeq database. We expect that in the fox transcriptome these regions will each be represented by an isogroup with only one isotig assigned to it.

Also, not all genes that are transcribed are described in the databases we used. The genes not in public databases are expected to be genes which would have the more rare, and less covered sequences, and subsequently less complex isogroups. This low level of expression would imply that they would have been less likely to be found and described previously. This may explain why only 28.31% (14,347) of all isogroups with a single isotig were successfully aligned using BLASTX. At the same time, 77.08% (6,960) of isogroups containing more than one isotig (9,030) yielded a hit to the SwissProt database.

The completeness of the fox transcriptome was tested by mapping fox reads combined variously into datasets of different sizes against the dog RefSeq database (Figure 2). One striking result of this experiment was the identification of a very similar number of dog gene orthologs in transcriptomes from tame and aggressive samples (13,618 and 13,855 genes, respectively). The total number of genes identified in both transcriptomes was only approximately 5% larger than a number of genes identified in individual samples (Figure 2). These data demonstrate that, without increasing the depth of sequencing by at least an order of magnitude, it is unlikely that a significantly higher number of rare transcripts would be identified in these transcriptome libraries. The number of genes identified, in this study, from fox pre-frontal brain transcriptome is similar to that identified from mouse cortex (14,787 genes at day E18 and 15,423 genes at day P7, respectively) [40].

For each accession number in the dog RefSeq database to which at least one fox read mapped, we estimated the breadth of coverage of dog transcripts by fox reads. Figure 3 demonstrates the bivariate coverage of identified transcripts: roughly 75% of dog transcripts had less than 90% coverage and ~25% of dog transcripts had coverage over 90%. We examined the depth of coverage of dog transcripts with relatively low coverage. The slight rise in the middle of the graph, from around 40-60% of coverage contains, as expected, mainly transcripts with a low depth of coverage, but it also contains a significant portion of the transcripts, responsible for the rise above a simple fit curve, that have a large number of fox reads mapped to them. It suggests that in many cases it is not the depth of coverage that is limiting the breadth of coverage. The transcripts in the canine RefSeq database can originate from different tissues and it is likely that for some genes, brain transcripts only partially overlap with transcripts from other tissues. Possible sequence differences between canine and fox transcripts would also reduce the coverage. The breadth of coverage of dog transcripts mapped by reads from two individual samples or from both samples together was almost identical. These data again suggest that a moderate further increase in the amount of sequencing data is unlikely to increase the breadth of coverage of dog transcripts.

The dog is the closest relative of the fox with a sequenced genome [41]. The dog and fox, however, have very different karyotypes, the dog having 78 mostly acrocentric chromosomes, whereas the red fox has 34 metacentric chromosomes [42,43]. The cytological relationship between the dog and fox genomes is well understood: cytogenetic methods [44-47] and alignment of the fox meiotic map against of the dog genome [34] have demonstrated that most fox chromosomes correspond to two or three canine chromosomes. The canine genome sequence assembly CanFam2 was used to localize fox sequencing reads and contigs in the dog genome. The approximate location of corresponding regions in the fox genome could be then inferred using the comparative dog-fox genetic and cytogenetic maps.

Mapping fox reads to the canine genome allowed the sequence divergence between the dog and fox genomes to be estimated. One sequence difference (single nucleotide difference, small indel, or difference in 2-3 adjacent nucleotides) between fox and dog was identified per 129 mapped bases. The number of differences between fox chromosomes was one difference per 1,309 mapped bases. The level of divergence between fox and dog coding regions is comparable with the level of divergence between sheep and cattle [48].

Mapping fox reads against both the canine genome and the assembled fox transcriptome sequence identified 30,491 high-confidence fox-specific SNPs. The filtering criteria were established by Sanger resequencing of SNPs with different numbers of reads per allele. The number of SNPs identified in this study significantly exceeded those identified in the bank vole transcriptome (19,114 SNPs), although filtering parameters in our study (minor allele present in at least three reads in one individual) significantly exceed the filtering parameters used in the vole transcriptome study (each allele present in at least two reads) [14]. These comparisons demonstrate that a greater depth of transcriptome sequencing enables the discovery of a large number of SNP's with higher confidence.

Our filtering parameters yielded 100% confirmation of the polymorphisms in the validation study (Additional file 5, Table S2a,b) but differences in individual genotype (homozygosity vs heterozygosity) identified by 454 sequencing were different from Sanger sequencing in some cases. To validate SNPs by Sanger sequencing, we used cDNA prepared from exactly the same RNA samples which were used for 454 sequencing (Methods section; SNP validation). Therefore, we expect allele specific bias to be equal in the samples used in both 454 and Sanger sequencing experiments. All SNPs for which the second allele in the same sample was not identified by 454 sequencing were among SNPs with the lowest read coverage depth.

Over 90% of SNPs from those with the lowest sequence coverage included in the validation study (minor allele present in at least three reads between the individuals) were also validated by Sanger sequencing. This filter parameter is still stricter than that in the bank vole study [14]. Therefore, for a small risk of false positives, we can also use some of these lower confidence SNP's to increase the total set of SNPs by about 50% to around 45,000 SNPs. Taking into account the significant cost difference between 454 and Sanger technologies, our data suggest that, at the small cost of obtaining a relatively small number of false polymorphisms, SNPs from this category would be worth including in a SNP array. A small number of false SNPs can be easily detected during post-processing analysis of genotyping calls and filtered out prior to further analysis. For regions in the fox genome associated with traits of interest it would also be useful to invest in validation of SNPs represented by an even lower number of reads. Although the false positive rate for these SNPs is expected to be higher, validation experiments can still provide a significant number of SNPs for these critical regions. The 454 technology has been successfully used for developing SNP resources for multiple organisms with limited genomic resources [49-51].

Initially, a large number of SNP's that mapped to the canine X chromosome were heterozygous in at least one male individual. To test whether this represented errors in our data or in the assembly of the canine X chromosome, the contigs bearing these SNPs were mapped onto the Human X chromosome assembly. All fox contigs containing SNPs that mapped to both the human and the dog X chromosome outside of the recognized pseudoautosomal region were homozygous for each individual fox. To gain a better understanding of the high heterozygosity of SNPs assigned to CFAX, we mapped approximately 100 bp region surrounding 50 randomly selected SNPs that were reported as heterozygous on the X back to the dog genome. We found that, in 34% of cases the area surrounding the SNP could not be located unambiguously to the X, or to a single area on the X; 50% of SNPs had high homology to the X but also had significant homology with another area of the genome. This casts doubt on the X chromosome SNPs showing heterozygosity in our male individuals. We subsequently filtered out all SNPs on the CFAX that were not in the pseudoautosomal region and were heterozygous in either individual.

Of all the fox high-confidence SNPs identified 97.8% were localised in the dog genome, and the inferred location in the fox genome was identified for 96.3% (Additional file 4, Figure S3; Figure 4). Heterozygosity of SNPs in the two fox samples was compared and several continuous regions along fox chromosomes where SNPs were fixed for opposite alleles between tame and aggressive sample were revealed. These regions warrant investigation in a larger number of samples from tame and aggressive fox strains for evaluation of putative selection sweeps. One extended region of reduced heterozygosity in the tame sample was observed in a region of VVU12 corresponding to part of a previously identified QTL interval [32]. Overall, the number of high confidence SNPs and their locations on fox chromosomes indicated that now we have a set of SNPs sufficient for genome wide mapping in these populations.

Detailed evaluation of the location in the dog genome of a randomly selected set of 100 fox SNPs revealed that approximately 77% of SNPs are located in transcribed regions and distributed approximately equally between coding regions and UTRs. Approximately 9% of SNPs are located in introns and 14% in non-coding regions. Similar results were also identified in the bank vole transcriptome study [14]. As has become apparent recently, the mammalian transcriptome is more complex than previously recognized [52-54].

Comparison of gene expression between the two fox samples revealed only a relatively small number of genes whose expression level differed by two-fold or more (355 genes p < 0.05). The biofunction groups over-represented in the set of differentially expressed genes differed between the two samples. Genes associated with neurological diseases were over represented in the tame sample. Interestingly, the second biofunction group over-represented in the aggressive sample represents genes associated with cardiovascular diseases. The links between the cardiovascular system and behavior have been increasingly recognized recently [55-58].

To validate differences in gene expression that were identified by transcriptome sequencing, we selected a subset of nine genes for expression analysis by RT-qPCR. Exactly the same two RNA samples (one from a tame and one from an aggressive fox) which had been used for transcriptome sequencing were used for RT-qPCR validation experiments. Eight of nine differentially expressed genes selected for the validation study showed differences in expression in the same direction by both methods. All genes that were validated by RT-qPCR showed differential expression between the two samples in transcriptome analysis with p < 0.04. Although the consistent validation by RT-qPCR of results obtained by transcriptome analysis increases confidence in the observed differences in expression between the two samples, it will clearly be necessary to test these observed differences in a larger set of samples before coming to conclusions regarding biological relevance. Among the genes that were validated by RT-qPCR, *HTR2C *is particularly noteworthy. *HTR2C *plays an important role in serotonergic and dopaminergic signalling [59-61]; and is differentially expressed in specific brain regions of two strains of rats - one of which was selected for and the other against aggressive behavior [62]. Increased level of *HTR2C *mRNA in the frontal cortex and hippocampus of tame rats was observed [62]. Notably, the direction of difference in *HTR2C *expression between the tame and aggressive fox individuals in the present study was consistent with that between rat strains in the Popova *et al*. study. None of the genes reported in other studies [63-65] investigating comparative gene expression in canid brain samples were significantly different between the two fox samples in the present study.

Further resequencing and deeper analysis of brain transcriptome, including identification of non-synonymous mutations, comparison of splice variants between tame and aggressive samples, and evaluation of differentially expressed genes in larger independent sample sets (biological replicates) will be necessary to characterize critical differences in these fox strains associated with markedly different behavioral phenotypes. In any experimental system there will be a certain amount of noise that might stem from a variety of sources. These biological replicates would let us explore the differences in the underlying biological system we are interested in and validate the differences in gene expression.

## Conclusions

Using new sequencing technologies it is now possible to develop genomic resources for species without a sequenced genome. That dramatically facilitates the study of interesting but previously inaccessible biological phenomena and helps to establish new animal models for genetic research. Overall, transcriptome sequencing from the prefrontal cortex of two fox individuals identified over 30,000 high-confidence fox-specific SNPs, fox orthologs of over 14,000 dog genes, and yielded new insights into potentially important differences in expression of genes in the pre-frontal cortex between tame and aggressive foxes.

## Methods

### Brain samples

Fox samples were collected at the experimental farm of the Institute of Cytology and Genetics in Novosibirsk, Russia. All animal procedures complied with standards for humane care and use of laboratory animals by foreign Institutions. Samples of pre-frontal cortex were collected into RNAlater (PE Biosystems, Foster City, CA) and stored according to the manufacturer's protocol, from two 7 month old male foxes (one from the tame and one from the aggressive population) and subsequently used for transcriptome sequencing.

### cDNA library preparation and sequencing

Total RNA was extracted using TRIZOL reagent (Invitrogen, Carlsbad, CA). cDNA libraries were prepared at Roche Life Sciences facility using Roche cDNA Rapid Library Preparation protocol (Method Manual, Roche, 2009) cDNA sequencing experiments require the conversion of mRNA into cDNA prior to preparation with the GS Rapid Library Prep Kit. Utilizing the cDNA Synthesis System Kit from Roche, transcription of RNA into cDNA is performed using 200 ng of RNA as starting material (OD 260/280 ≥ 1.8). mRNA is isolated and then treated with zinc chloride to fragment the mRNA into the desired 450 bp range. The sequencing read length is determined by the fragmentation of the RNA. RNA size varies.

Random hexamer primers were used, comprising single-stranded DNA containing every possible 6-base combination, to enable hybridization anywhere on the RNA. Once randomly fragmented, cDNA first-strand synthesis is primed utilizing the random hexamers, which diminishes the priming of the 3' poly(A) tail; it is then followed by second-strand synthesis. From this hybridization, reverse transcriptase utilizes the double-stranded sequence as a primer to start translation. The double-stranded cDNA is used directly as starting material for rapid library preparation, yielding blunt-ended cDNA with the addition of the overhanging A. The resulting cDNA fragments are then polished and prepared for 454 Adaptor ligation. Once ligated, the DNA is ready for emulsion PCR (emPCR TM). 454 pyrosequencing is then performed using standard GS FLX Titanium series reagent kits.

### 454 GS FLX Titanium sequencing

Amplified products were sequenced at Roche Life Sciences facility using the Roche 454 FLX Titanium platform. Samples were sequenced in parallel in 6 runs. Data for 5 half full runs for tame individual and 6 half full runs for aggressive individual were obtained.

### Post-processing of sequencing reads

Image analysis, signal processing and data filtering were performed using the standard 454 Software with default settings. 454 reads were first quality filtered with standard parameters and raw reads were cleaned from adaptor sequences. Files containing 454 reads and their quality scores are available from the National Center for Biotechnology Information (NCBI) Sequence Read Archive [SRA029285.1]. All fox reads that passed quality filtering were retained for subsequent analysis (Table 1).

### *De novo *assembly of fox reads

Fox sequencing reads were assembled using the 454 GS *De Novo *Assembler software (March 2010, R&D release) with the transcriptome (-cdna) option activated. The reads were assembled in three combinations, resulting in three separate assemblies of fox transcriptome: i) assembly of all sequencing reads from both samples; ii) assembly of sequencing reads from the sample of tame fox; iii) assembly of sequencing reads from the sample of aggressive fox. All three assemblies were generated using the same parameters: minimum overlap length of 40 bp, minimum identity of 90%, with cDNA mode set to true. A trimming FASTA file, which includes polyA sequences of 5 bp, 10 bp and 20 bp in length, was used in conjunction with the vector-trimming option (-vt) of the assembler software, in order to trim off the polyA tails among the sequencing reads prior to assembly.

For each assembly, GS *De Novo *Assembler generated a set of output files including: i) "454NewblerMetrics.txt", which reports numerous overall assembly metrics; ii) "454AllContigs.fna", which contains the nucleotide sequences of the assembled contigs generated by the assembly process; iii) "454Isotigs.fna", which contains the nucleotide sequences of the assembled isotigs and their corresponding isogroups, as generated by the assembly process.

Reads were analyzed into contigs and singletons, to generate sets of isotigs, and to classify isotigs into isogroups. In brief, a contig corresponds to the consensus sequence of a self-consistent set of overlapping reads, as determined by the algorithmic steps of the 454 GS *De Novo *Assembler software, which include pairwise overlap, multiple sequence alignment, detangling and basecalling. An isogroup is a collection of contigs containing reads that imply connections between them. An isotig is meant to be analogous to an individual transcript. Different isotigs within a given isogroup can be inferred to be splice-variants. The reported isotigs are the putative transcripts that can be constructed using overlapping reads provided as input to the assembler.

### BLAT of fox contigs to the dog genome

All contigs at least 100 bp long were located on the dog genome (CanFam2) by BLAT and the result yielding the best score was kept for each. The results (.psl files) from using BLAT to locate fox contigs on the dog genome, and the output file from a similar BLAT run using only contigs with sequence differences between foxes, were uploaded to the web as custom tracks on the UCSC genome browser to visualize their locations on the dog genome. To identify fox X-chromosome sequences, all contigs at least 100 bp long were also mapped to the human genome by BLAT: those that mapped over more than 50% of their length to the human X, and also mapped to the dog X were scored as located on the X chromosome with high confidence.

### BLASTX of fox isotigs to the protein database

BLASTX was run, comparing the isotigs.fna file to the SwissProt database. An e cutoff of 1e-05 was used. When more then one entry in the SwissProt database was hit, the one with the best e-value was used to assign isotigs to genes. In the case of a tie the one listed first in the file was used.

### Mapping sequencing reads to the canine RefSeq database

Cleaned sequencing reads from the tame and aggressive individuals were mapped in separate runs to the canine RefSeq database using 454 GS Reference Mapper (Version 2.3), with a threshold of 95% identity over 90% of length, using the cDNA mode. For each mapping, GS Reference Mapper generated a series of output files including: i) "454RefStatus.txt", which reports the statistical information on the number of reads mapping to each reference sequence; ii) "454GeneStatus.txt", which reports statistics on the number of reads mapped exclusively to each gene; iii) "454AllDiffs.txt", which contains the list of variations (of at least two reads) relative to the reference sequence or to other reads aligned at a specific location; iv) "454HCDiffs.txt", which contains the list of high confidence variations relative to the reference sequence or to other reads aligned at a specific location. The canine RefSeq database was downloaded from NCBI http://www.ncbi.nlm.nih.gov/nuccore/limits[66] on June 15, 2010, using the search parameters: Organism=Canis lupus familiaris, Database = RefSeq, Molecule=mRNA. 33,476 sequences representing 19,859 genes were downloaded. Because of redundancy in the RefSeq data set, caused by splice variants of genes having multiple accession numbers, many of the fox reads mapped to multiple references and were not reported in the 454RefStatus.txt file. To solve this problem, a rename file was created to assign multiple dog splice variants to the same gene using the -accno option of GS Reference Mapper. The 454GeneStatus.txt file was then used to determine the number of fox sequencing reads corresponding to each gene. To estimate the total percentage of genes from the dog genome in our data set, we calculated the percentage of genes from the canine RefSeq database that were represented by at least one sequence in our set.

Breadth of coverage of dog transcripts by fox reads was calculated as percent of homologous dog transcript covered by at least one uniquely mapped read as reported in the file 454RefStatus.txt.

### Mapping fox sequencing reads against the canine genome sequence assembly

Fox sequencing reads were mapped to the repeat-masked, annotated canine genome sequence assembly (CanFam2, UCSC) with GS Reference Mapper (Version 2.3) at a threshold of 95% identity over 90% of length, using cDNA mode. Information about single nucleotide differences, indels, and sequence differences involving two or three adjacent nucleotides was extracted from GS Reference Mapper file "454AllDiffs.txt". To estimate the divergence of the dog from the fox, the number of nucleotide differences reported in the file "454AllDiffs.txt" and identified between dog and fox for which fox reads did not differ from each other, was compared to the number of nucleotide differences observed between fox reads.

Fox-specific nucleotide differences were extracted from the output file "454HCDiffs.txt" and filtered to create a set of higher confidence differences. Retained SNPs were selected by the following criteria: the minor allele had to be present in at least 25% of the sequencing reads per sample, each SNP allele had to be present in at least three reads per allele for an individual, and SNPs assigned to CFAX outside of pseudoautosomal region were homozygous for each individual.

### Identification of fox SNPs using the fox assembly as reference

The set of contigs over 100 bp from the assembly of all reads (All together) was used as a reference to map all sequencing reads using GS Reference Mapper (Version 2.3) with default parameters using cDNA mode. The 454HCDiffs.txt file was used to find sequence differences between and within foxes: the information about single nucleotide differences, indels and 2-3 nucleotide polymorphisms (two or three adjacent nucleotides that differ from the reference sequence) was extracted from "454HCDiffs.txt" file. Single nucleotide differences were filtered to remove (i) those for which the minor allele in an individual accounted for less than 25% of the sequencing reads and (ii) those with less than three reads for an allele for an individual, (iii) those that were not SNPs, (iv) SNP's assigned to the canine X chromosome were further filtered to remove SNPs which had apparent heterozygosity in either male individual, and assigned to the part of CFAX outside of the recognized pseudoautosomal region. The list of differences was also classified according to heterozygosity/homozygosity in each individual. The contigs were assigned to the dog genome by BLAT and the results were used to locate the fox SNPs. This set of SNPs and the set of SNPs identified by mapping fox reads to the dog genome were combined and the duplicates between the sets were removed. The inferred location of these SNPs on fox autosomes was assigned using the comparative fox/dog map.

### SNP validation

To establish parameters for selecting high-confidence SNPs identified by both methods - (i) mapping fox reads against the dog genome; and (ii) mapping fox reads against fox contigs - we selected a subset of SNPs for validation by Sanger sequencing. Three sets of SNPs for validation experiments were generated using the following parameters: 1) the minor allele had to be represented in at least 25% of the sequencing reads per sample and each SNP allele had to be represented in more then three reads per allele for an individual; 2) the minor allele had to be represented in at least 25% of the sequencing reads per sample and each SNP allele in at exactly three reads per allele for an individual; 3) the minor allele had to be represented in at least three reads between two individuals.

All sequencing reads corresponding to selected SNPs were extracted and aligned manually using Sequencher^®^ 4.2.2 Software (Gene Codes Corporation, Ann Arbor, MI). Assembled contigs were used for primer design. Primers were designed using Primer3 to amplify across the SNPs (Additional file 6, Table S3).

PCRs were performed using fox cDNA as a template. Same RNA samples which were used for 454 sequencing were converted into cDNA and used in SNP validation study. Fox cDNA was synthesized as described in the section "Real-Time PCR". PCRs were performed using 0.5 μl of cDNA from the standard cDNA reaction recommended by Thermoscript kit (Invitrogen, Carlsbad, CA). PCR reactions were performed in 25 μl containing 10mM of each primer and 1× GoTAQ (Promega, Madison, WI) master mix. Standard PCR conditions were used: an initial 2 min denaturation at 96°C; then 30 cycles of 96°C (20 seconds), 58°C (20 seconds), 72°C (20 seconds); and a final extension step at 72°C for 10 minutes. PCR fragments were purified using Qiagen PCR purification kit (Qiagen, Valencia, CA) and sequenced from both directions. Sequencing was performed on an ABI3730 Genetic Analyzer (PE Biosystems, Foster City, CA) at Cornell sequencing facility. Sequencing reads were assembled and compared to the 454 reads using Sequencher®.

### Analysis of SNP distribution among coding and non-coding regions

100 SNPs were chosen at random to estimate the overall percentage of SNPs in coding regions. Each of the 31,264 SNPs from the combined unique set were assigned a random number using the random function of FileMakerPro, version 7 (FileMaker Inc., Santa Clara, CA), and the 100 with the lowest number were chosen for further characterization. A short sequence (30-62 bp) surrounding the polymorphism was localized to the dog genome using BLAT, and the "RefSeq Genes" track and the "Non-Dog RefSeq Genes track" were used to judge if the location of the polymorphism was located in a coding region, in an intron that is between coding exons, in an area where the gene untranslated region (utr) is likely to be (either in a known utr, or in the boundaries of utr's from other species), in inter-gene space, or non-locatable.

### Comparison of gene expression between two fox samples using transcriptome sequencing

To estimate the difference in gene expression between the two fox samples, the number of hits to a given RefSeq gene for each individual was first normalized to the total number of reads for the same individual, to thus reduce possible bias introduced by an overall greater number of total reads for one individual versus the other. To ensure that this normalization did not inappropriately distort the data, MA plots [67] were examined pre and post normalization to compare the patterns of data distribution (Additional file 7, Figure S4). The difference in this normalized count between individuals, was used as an estimate of expression difference between the individuals for the relevant gene. A Z-test was run on the data and a two-tailed p-value was computed for each gene. The Z-score (λ-value in [68]) was computed using test statistics defined by equation (2) in Reinartz *et al*. [68]. The p-value (two-sided) was calculated in Microsoft Excel using the formula: p = 1-|(NORMDIST(λ)-NORMDIST(-λ))|.

### Real-Time PCR

A subset of genes with differences in expression between the two samples was selected for quantitative Real-Time PCR (RT-qPCR) validation. Sequencing reads corresponding to each gene were extracted and assembled manually using Sequencher^®^ 4.2.2 Software (Gene Codes Corporation, Ann Arbor, MI). Assembled contigs were aligned against the canine genome (CanFam2) using BLAT (UCSC, CA). Intron-exon boundaries within fox contigs were identified by comparison of fox contigs to the corresponding canine genes. Primers were designed to amplify across intron-exon boundaries to minimize amplification of gDNA (Additional file 8, Table S4), using Primer3 (Broad Institute, Boston, MS) with parameters optimized for RT-qPCR conditions (primer size: min 18 bp, opt 20 bp, max 23 bp; primer Tm: min 58°C, opt 60°C, max 62°C; product size: 75-150 bp).

Fox cDNA was prepared using Thermoscript kit (Invitrogen, Carlsbad, CA). Aliquots of the same RNA samples that had been previously prepared for the transcriptome sequencing experiment were used for RT-qPCR experiments and SNP validation study. cDNA was synthesized using total RNA (1 mkg per reaction) and oligo dT primer according to the manufacture protocol (Invitrogen, Carlsbad, CA). RT-qPCR was performed using SYBR Green Fast (PE Biosystems, Foster City, CA) on an ABI machine 7500 Fast (PE Biosystems, Foster City, CA) following the manufacturer's instructions. RT-qPCR reactions were performed in 20 μl volumes containing gene-specific primer pairs (0.15 or 0.3uM), 2μl of cDNA and 10 μl of SYBR Green Fast (PE Biosystems, Foster City, CA). Reactions were performed in triplicate. Primer concentration was optimized for each primer pair. Fox gDNA and water were used as controls. All reactions were performed under standardized conditions: an initial denaturation at 95° (20 seconds); then 40 cycles of 95° (3 seconds), 60° (30 seconds); following by dissociation step: 95° (15 seconds), 60° (20 seconds), 95° (15 seconds), 60° (15 seconds). Results were analyzed using SDS1.4 software (PE Biosystems, Foster City, CA).

Relative quantification was performed using *HPRT1 *gene as an endogenous control. A genome-wide panel of canine reference genes has been evaluated by Brinkhof et al., (2006) [69]. This study advocated using ribosomal protein S19 (*RPS19*), ribosomal protein S5 (*RPS5*), b-2-microglobulin (*B2M*), and hypoxanthine phosphoribosyltransferase (*HPRT*) as reference genes for RT-qPCR. *HPRT *has been used as a reference gene for RT-qPCR in multiple canine studies [[70-74], and others ]. *HPRT *was also found to be one of the most stable reference genes in the cerebral cortex in rats [75]. *HPRT1 *primers were modified from dog-specific primers [71] using fox sequence (Additional file 8, Table S4). RT-qPCR reactions for target genes and *HPRT1 *were performed in different tubes. RT-qPCR for each tested gene and *HPRT1 *were run simultaneously on the same plate. Relative gene expression levels were calculated using 2^-ddCt^ method [76].

## Authors' contributions

Conceived and designed the experiment: GMA, AVKu, LNT, CT. Provided study animals and collected samples: LNT, INO, AVKh, RGG, AVV, DVS, SGS. Performed and analyzed transcriptome assembly: LL, JLJ. Mapped 454 reads to the dog genome: JLJ, LL. Defined gene content of the transcriptome: JLJ, LL, AVKu. Identified and validated SNPs: JLJ, LL, AVKu, RP, MMD. Analyzed expression difference between two samples: JLJ, LL, AVKu. Advised on bioinformatic and statistical analyses: LL, CT, QS, LP. Critically interpreted results: SVT, GMA, AVKu, JLJ, CT, LL. Wrote the paper: AVKu, JLJ, GMA, SVT, LL, CT. Revised the paper: GMA, AVKu, JLJ, SVT, CT, LL, LNT, AVKh, INO, RP. All authors read and approved the final version of the manuscript.

## Supplementary Material

Additional file 1 **Supplementary Figure 1. Visualisation of fox contigs on the UCSC genome browser**. A short region of canine chromosome 1 (CFA1; chr1: 91,191,500 - 91,674,000) (modified from UCSC genome browser output) with contigs from the silver fox transcriptome added as a User Supplied Track (highlighted region). Each of the 15 fox contigs aligned to this interval correspond to known RefSeq genes, two of which (TJP2 and FXN) are identified in both the Canine RefSeq and Non-Dog RefSeq database tracks, and several that are not in the Canine RefSeq, but are in other Non-Dog RefSeq databases. Click here for file

Additional file 2 **Supplementary Table 1. Putative homologs of canine genes identified in fox transcriptome**. Click here for file

Additional file 3 **Supplementary Figure 2. Examples of SNPs identified by aligning the fox transcriptome against the canine genome sequence assembly**. **(a) **an example of a fox vs dog polymorphism. The first row displays the canine sequence from CFA30: 11,097,645 to 11,097,706. Fourteen fox transcriptome reads are aligned to this canine sequence demonstrating a consistent transitional polymorphic difference (G<>A) between the dog and fox (boxed and highlighted nucleotide). **(b) **an example of fox vs fox polymorphism. The first row displays the canine sequence from CFA1:17,040,181 to 17,040,238. The first set of 10 fox reads, aligned immediately below the canine sequence, are identical to the canine sequence, except for an inserted C after the 13th nucleotide of the first such read, which is presumed to be a sequencing artefact. The second, lower set of 10 fox reads all differ from both the canine sequence and the first 10 fox reads by a transversional polymorphic difference (C<>A) at the 29th nucleotide position of the dog sequence (boxed and highlighted nucleotide) Click here for file

Additional file 4 **Supplementary Figure 3. SNP distribution and informativeness on fox autosomes**. Each fox autosome (VVU1 thru VVU16) is graphed to indicate the position and zygozity of SNPs detected by transcriptome analysis of one tame and one aggressive fox. The top row of lines indicates how the homologous canine chromosomes (CFAn) align to the specific fox autosome. The next row (cM) provides fox meiotic map distance information. The bottom row indicates estimated position in megabases, based on extrapolation from the canine genome sequence assembly. On the four central tracks, each SNP is represented by a single tick mark (+) for each individual (Aggressive or Tame), on either the heterozygous or homozygous line for that individual. A corresponding higher resolution view of portion of VVU12 is provided in Figure 5. Click here for file

Additional file 5 **Supplementary Table 2. Results of SNP validation by Sanger sequencing**. Three sets (**a**, **b**, **c**) of SNPs were selected for validation by the following criteria: for set (**a**) the minor allele had to be present in at least 25% of the sequencing reads per sample and each SNP allele had to be represented in more then three reads per allele for an individual; for set (**b**) the minor allele had to be present in at least 25% of the sequencing reads per sample and each SNP allele in at exactly three reads per allele for an individual; and for set (**c**) the minor allele had to be present in at least three reads between the two individuals. Validation was undertaken by Sanger sequencing SNP-specific amplicons amplified from aliquots of the same cDNA submitted for 454 transcriptome sequencing. For each SNP the allele(s) identified in the tame and aggressive samples by 454 sequencing are tabulated with the number of 454 reads supporting the allele(s), together with the allele(s) identified by Sanger sequencing. For SNPs for which an allele was identified in a sample by one method (454 or Sanger) but not the other, the alleles are indicated in bold font, and if the allele was not found by 454 sequencing the number of reads is zero. Click here for file

Additional file 6 **Supplementary Table 3. Primers for SNP validation experiment**. Forward and reverse primer sequences, designed using Primer3, are listed that amplify 51 fox target sequences, for validation of selected SNPs identified from fox transcriptome sequence analysis. Click here for file

Additional file 7 **Supplementary Figure 4. MA plots of the expression data before (a) and after (b) normalization for the higher number of aggressive sequences**. Before normalization (a) the median value (red line) exceeds zero (blue line) reflecting the overall higher number of reads from the aggressive individual. After normalization (b) the median is closer to zero, indicating that the normalization did reduce the initial bias, without distorting the data, but the median is now slightly below zero. The latter negativity of the median in the normalized graph represents a slightly higher proportion of tame reads that map to the dog RefSeq than do aggressive reads. As the reads were mapped to a different species' genome, and because only two samples were utilized it is not possible to know if this difference results from differences in expression, differences in sample quality or an artifact of dog vs fox sequence differences. Click here for file

Additional file 8 **Supplementary Table 4. Primers for RT-qPCR experiments**. Forward and reverse primers are listed that were used to amplify gene-specific fox amplicons by RT-qPCR to validate expression differences identified by transcriptome sequence analysis. Click here for file
