# Supplementary figures and images for: Sequence comparison of prefrontal cortical brain transcriptome from a tame and an aggressive silver fox (Vulpes vulpes)
Source: BMC Genomics. 2011 Oct 3;12:482. doi: 10.1186/1471-2164-12-482 (PMC3199282; doi:10.1186/1471-2164-12-482)

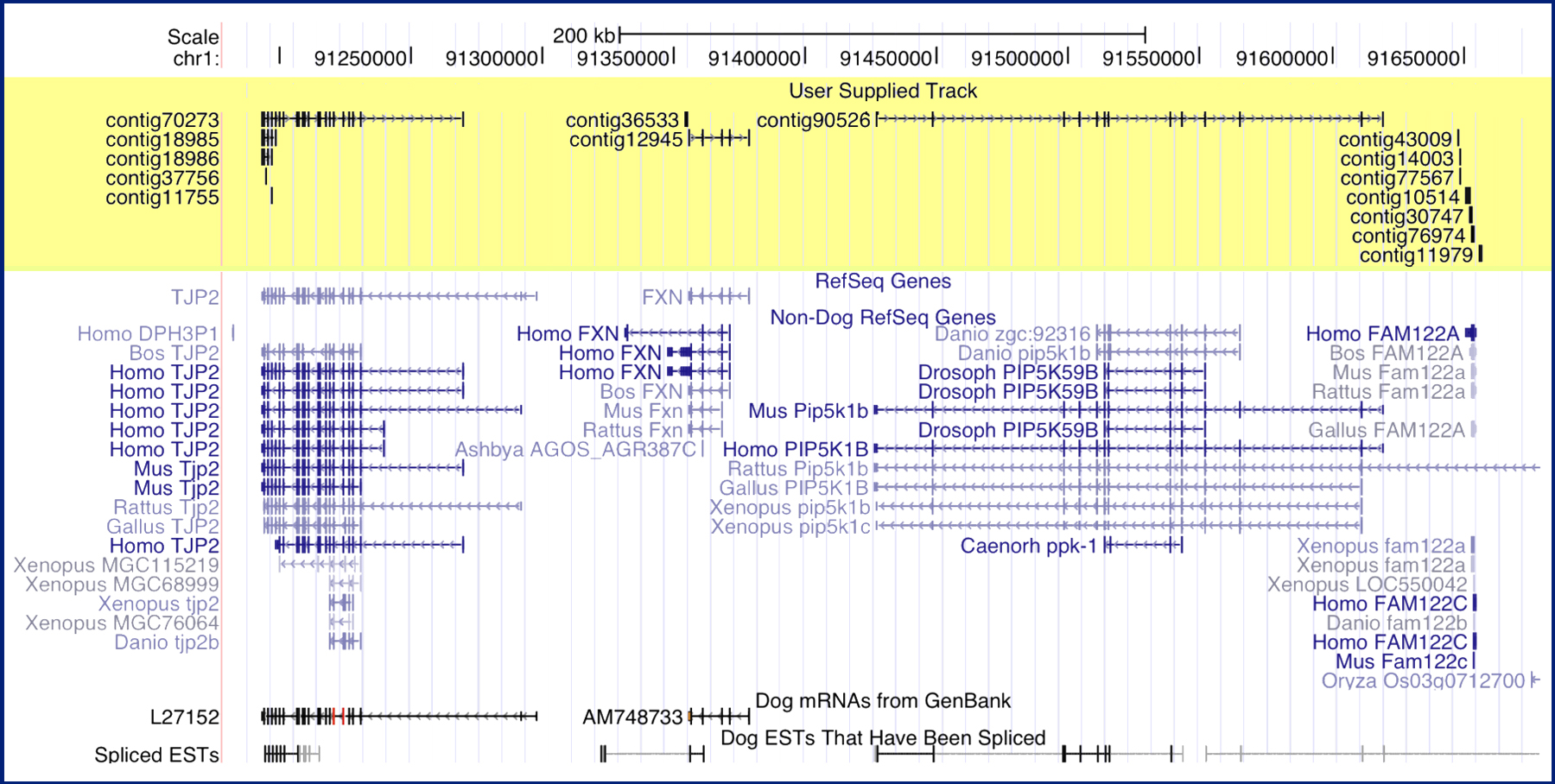

Supplement: Additional file 1 — Supplementary Figure 1. Visualisation of fox contigs on the UCSC genome browser. A short region of canine chromosome 1 (CFA1; chr1: 91,191,500 - 91,674,000) (modified from UCSC genome browser output) with contigs from the silver fox transcriptome added as a User Supplied Track (highlighted region). Each of the 15 fox contigs aligned to this interval correspond to known RefSeq genes, two of which (TJP2 and FXN) are identified in both the Canine RefSeq and Non-Dog RefSeq database tracks, and several that are not in the Canine RefSeq, but are in other Non-Dog RefSeq databases. [file 1471-2164-12-482-S1.JPEG]

Vvu1

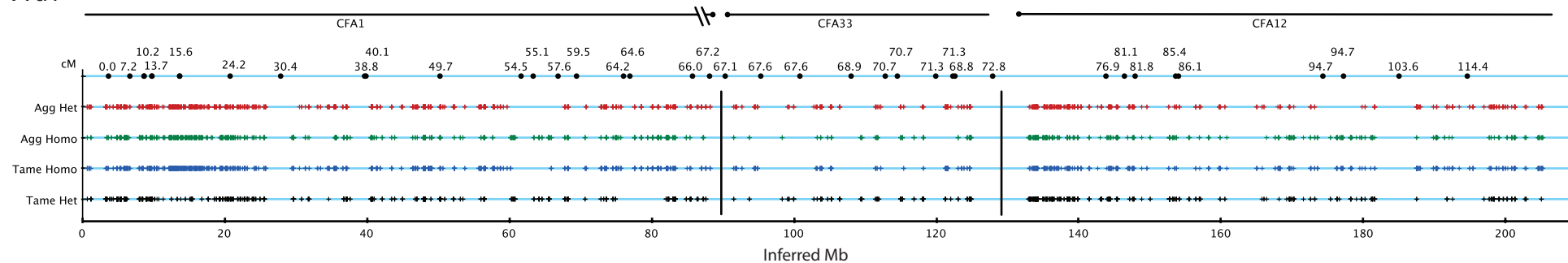

Vvu2

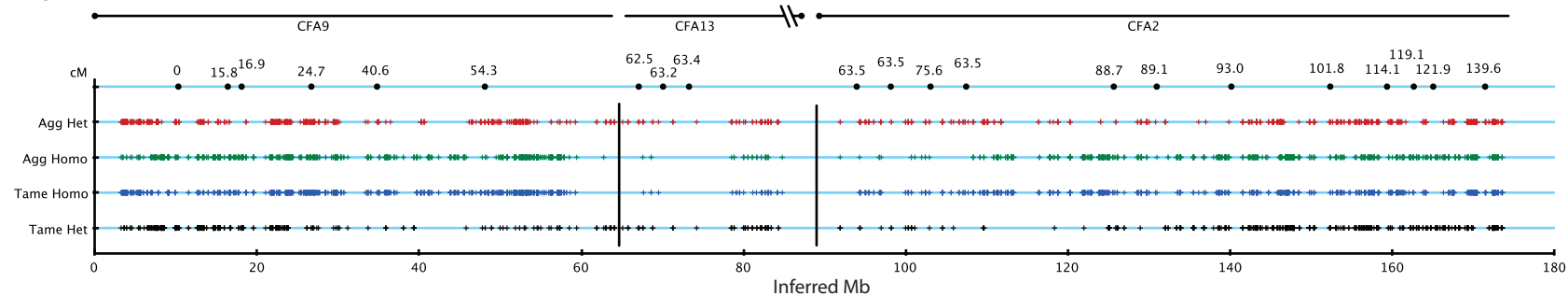

## Vvu3

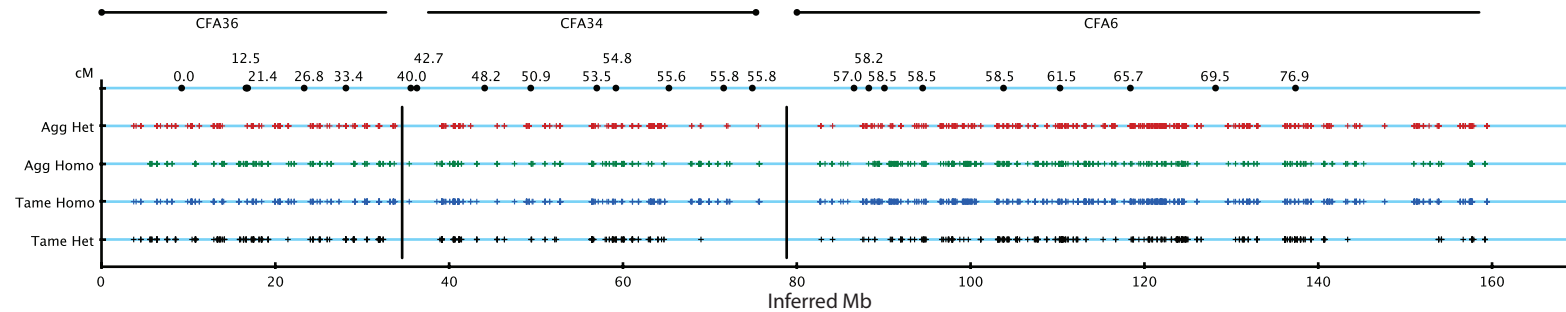

## Vvu4

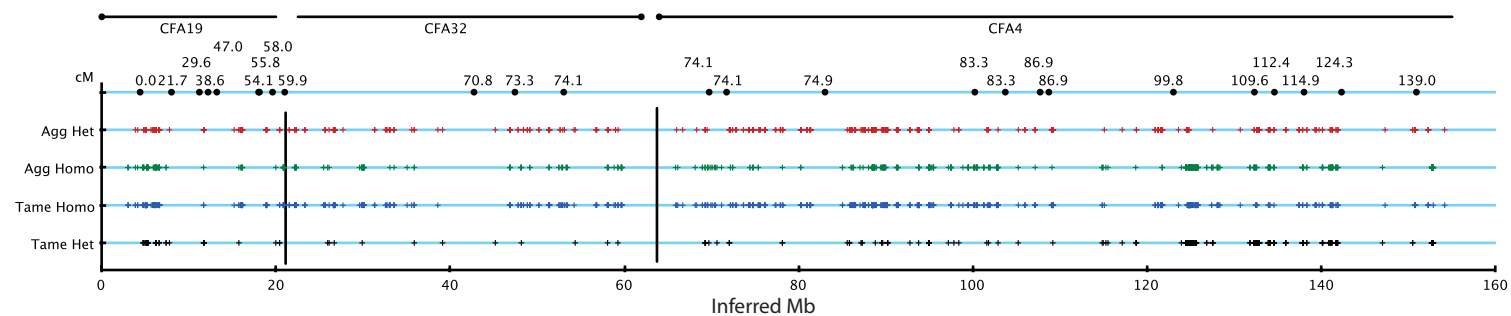

Vvu5

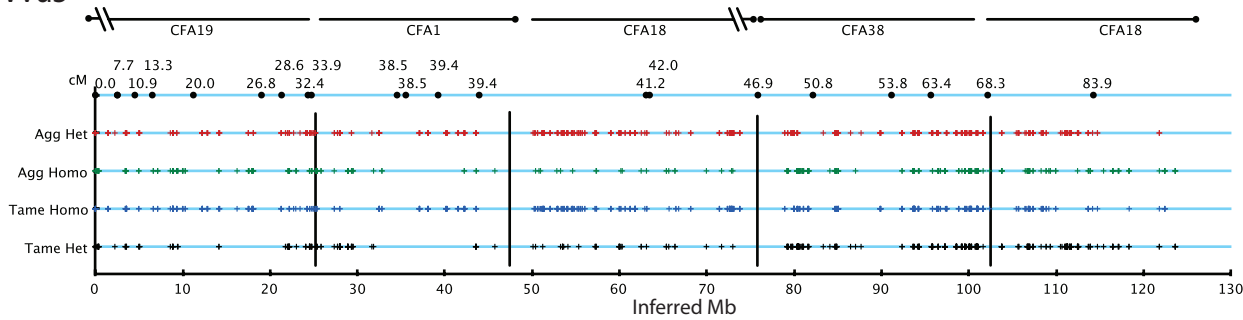

Vvu6

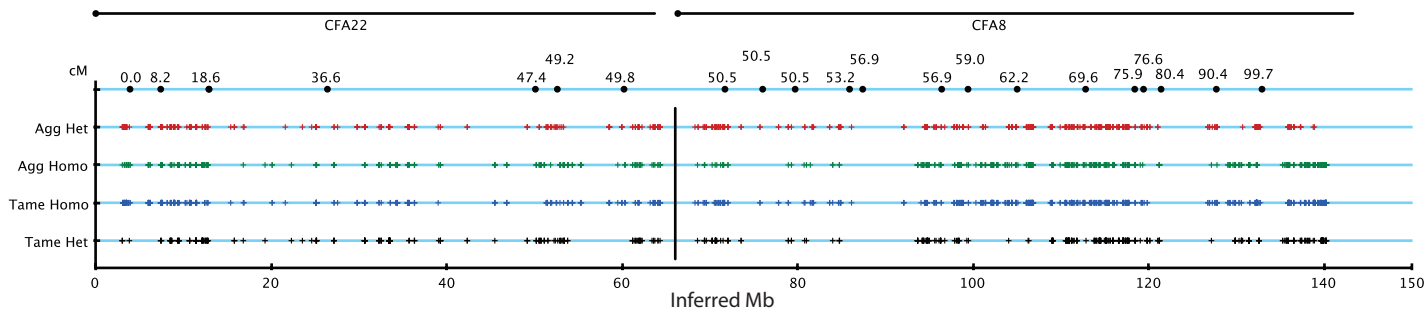

Vvu7

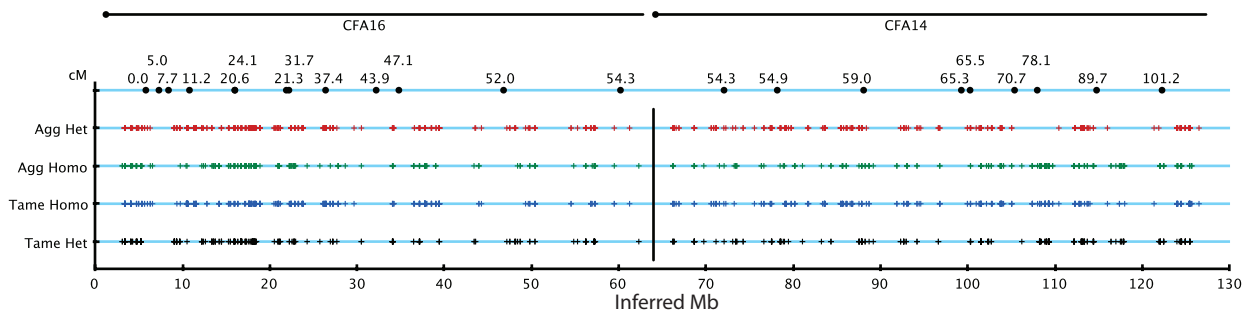

Vvu8

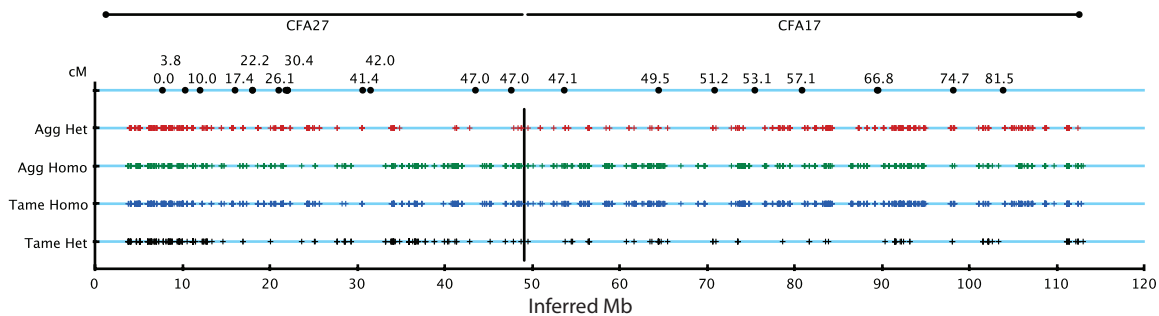

Vvu9

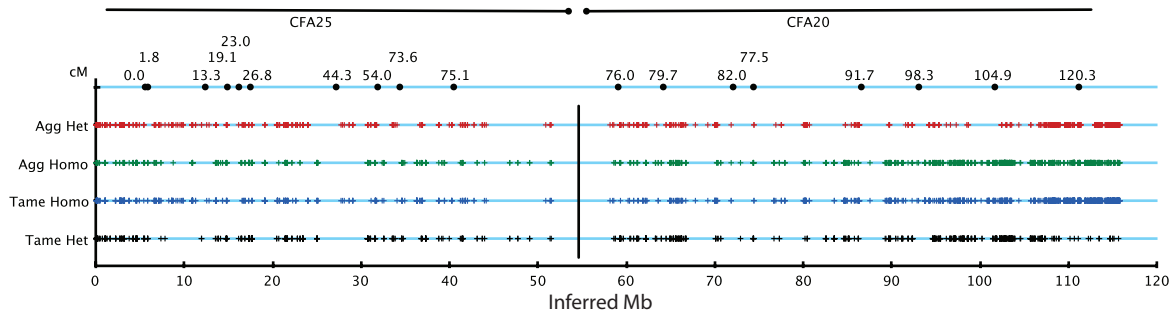

Vvu10

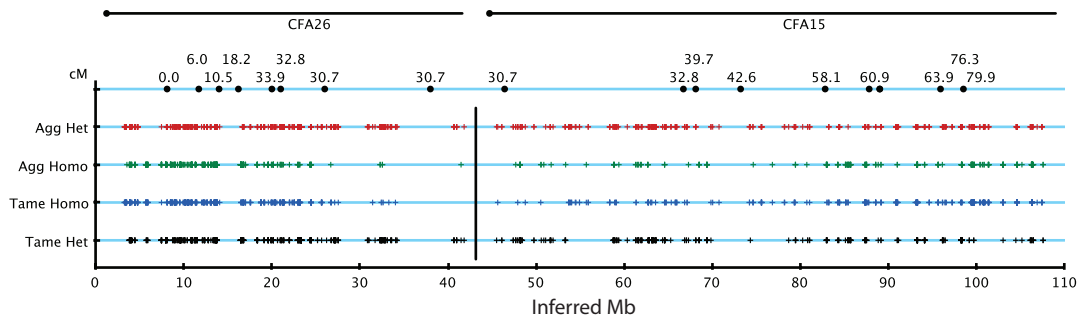

Vvu11

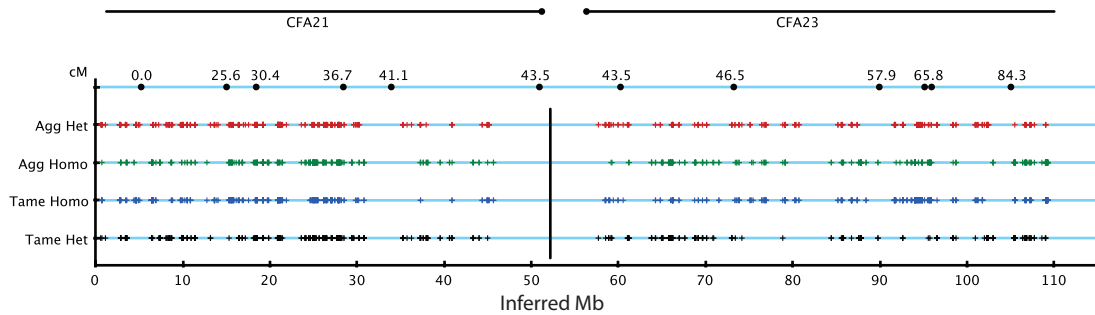

Vvu12

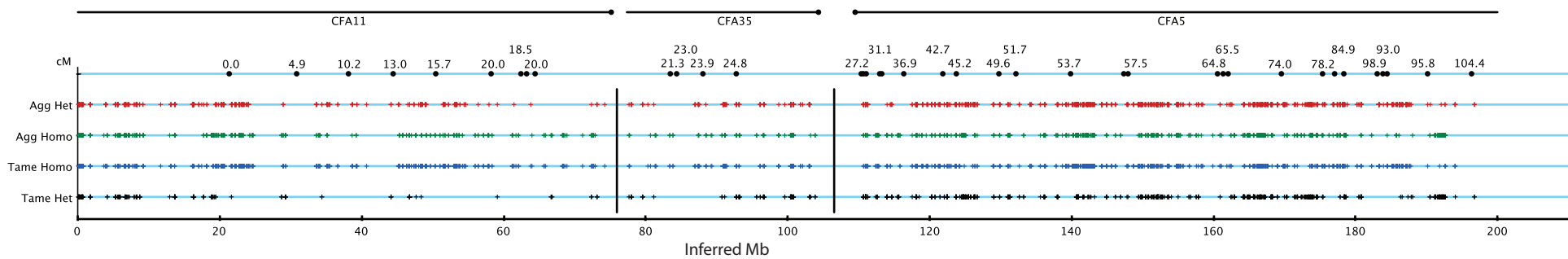

Vvu13

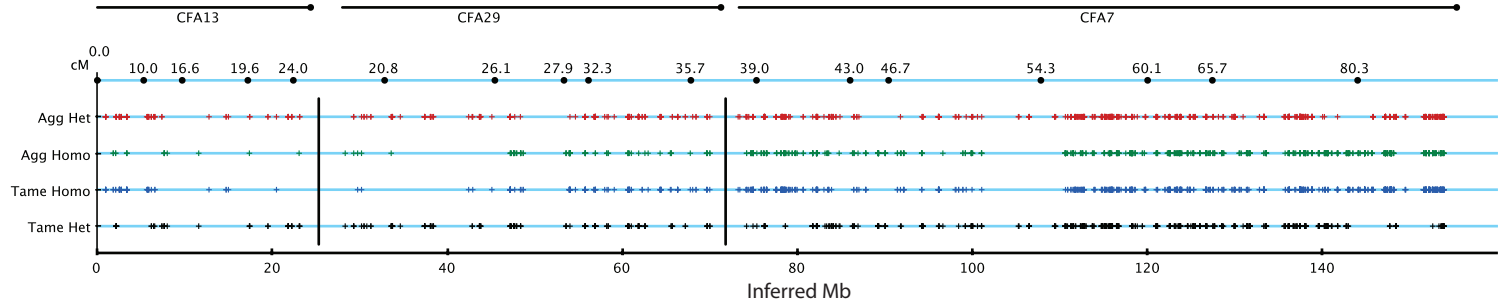

Vvu14

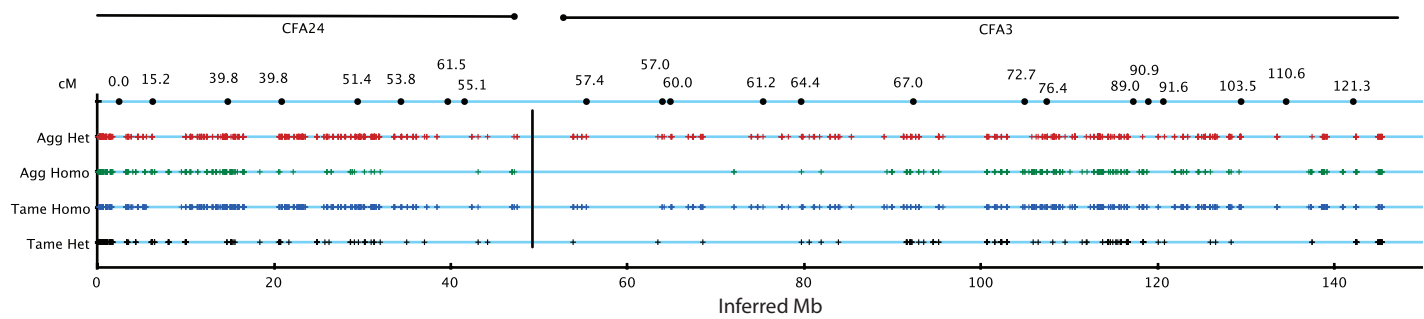

Vvu15

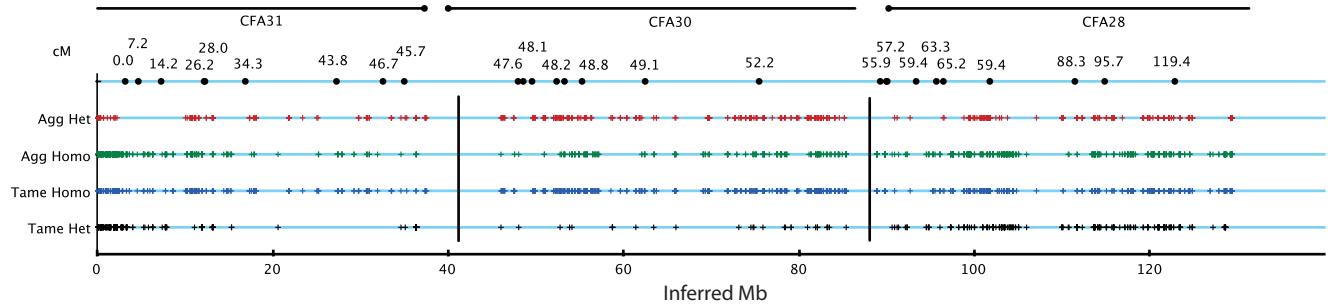

Vvu16

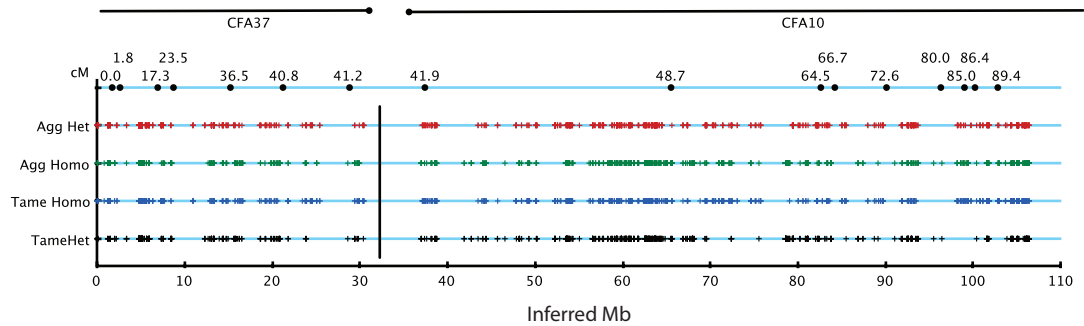

Supplement: Additional file 4 — Supplementary Figure 3. SNP distribution and informativeness on fox autosomes. Each fox autosome (VVU1 thru VVU16) is graphed to indicate the position and zygozity of SNPs detected by transcriptome analysis of one tame and one aggressive fox. The top row of lines indicates how the homologous canine chromosomes (CFAn) align to the specific fox autosome. The next row (cM) provides fox meiotic map distance information. The bottom row indicates estimated position in megabases, based on extrapolation from the canine genome sequence assembly. On the four central tracks, each SNP is represented by a single tick mark (+) for each individual (Aggressive or Tame), on either the heterozygous or homozygous line for that individual. A corresponding higher resolution view of portion of VVU12 is provided in Figure 5. [file 1471-2164-12-482-S4.PDF]

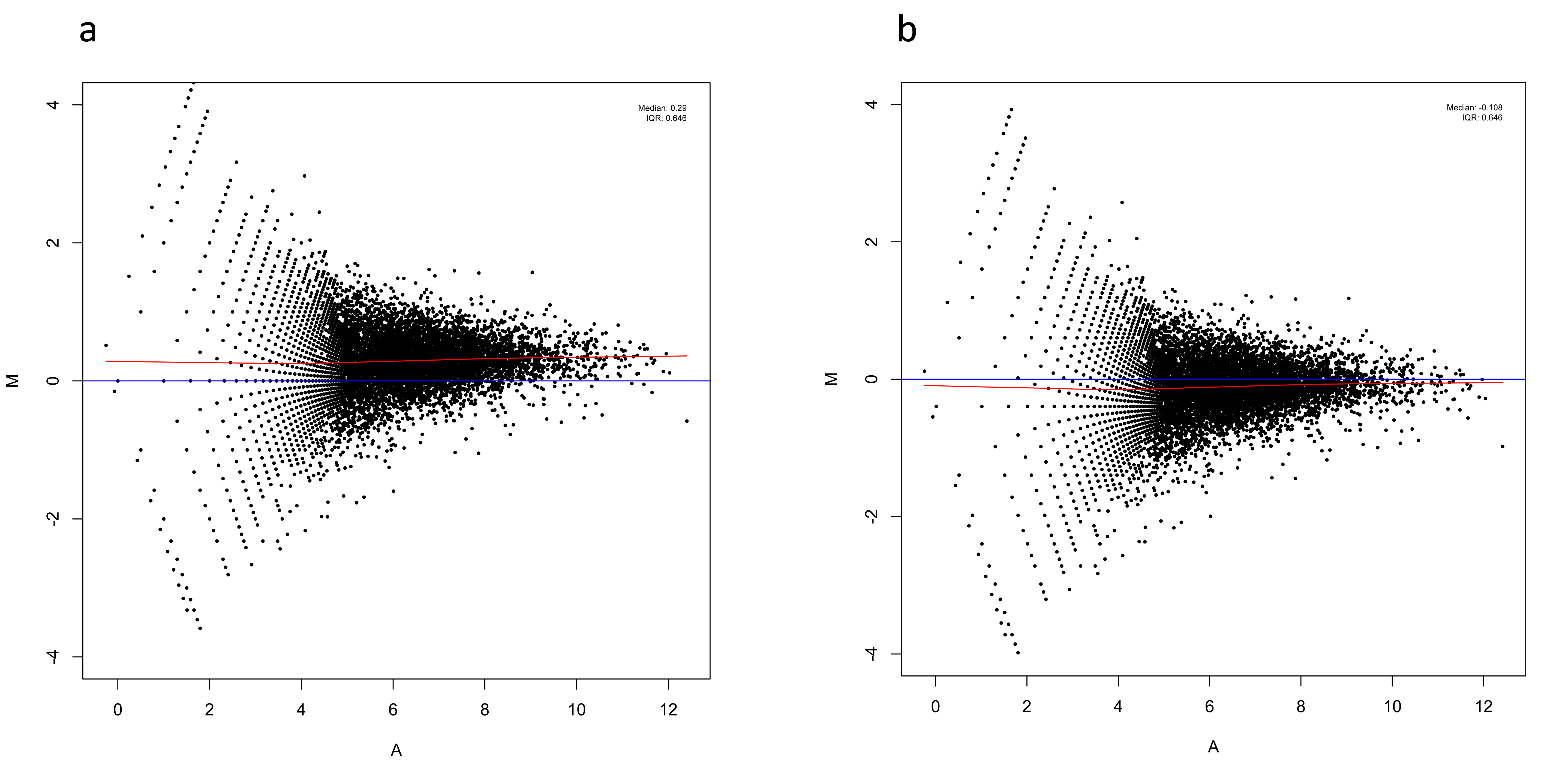

Supplement: Additional file 7 — Supplementary Figure 4. MA plots of the expression data before (a) and after (b) normalization for the higher number of aggressive sequences. Before normalization (a) the median value (red line) exceeds zero (blue line) reflecting the overall higher number of reads from the aggressive individual. After normalization (b) the median is closer to zero, indicating that the normalization did reduce the initial bias, without distorting the data, but the median is now slightly below zero. The latter negativity of the median in the normalized graph represents a slightly higher proportion of tame reads that map to the dog RefSeq than do aggressive reads. As the reads were mapped to a different species' genome, and because only two samples were utilized it is not possible to know if this difference results from differences in expression, differences in sample quality or an artifact of dog vs fox sequence differences. [file 1471-2164-12-482-S7.JPEG]
