# Supplementary material for: Sequence comparison of prefrontal cortical brain transcriptome from a tame and an aggressive silver fox (Vulpes vulpes)
Source: BMC Genomics. 2011 Oct 3;12:482. doi: 10.1186/1471-2164-12-482 (PMC3199282; doi:10.1186/1471-2164-12-482)
Supplement: Additional file 3 — Supplementary Figure 2. Examples of SNPs identified by aligning the fox transcriptome against the canine genome sequence assembly. (a) an example of a fox vs dog polymorphism. The first row displays the canine sequence from CFA30: 11,097,645 to 11,097,706. Fourteen fox transcriptome reads are aligned to this canine sequence demonstrating a consistent transitional polymorphic difference (G<>A) between the dog and fox (boxed and highlighted nucleotide). (b) an example of fox vs fox polymorphism. The first row displays the canine sequence from CFA1:17,040,181 to 17,040,238. The first set of 10 fox reads, aligned immediately below the canine sequence, are identical to the canine sequence, except for an inserted C after the 13th nucleotide of the first such read, which is presumed to be a sequencing artefact. The second, lower set of 10 fox reads all differ from both the canine sequence and the first 10 fox reads by a transversional polymorphic difference (C<>A) at the 29th nucleotide position of the dog sequence (boxed and highlighted nucleotide) [file 1471-2164-12-482-S3.PDF]

A

```
Cfa30                   11097645+ AGACACATTGTACCCGTATGCAGCATAGGCGGCGGCGGAGGCTGCTGCAGCCCCTGCTTTGG 11097706
                                                                *
GCT3NVP01DP281               145- AGACACATTGTACCCGTATGCAGCATAGGCAGCGGCGGAGGCTGCTGCAGCCCCTGCTTTGG 84
GCXRAE302GXVB6               222- AGACACATTGTACCCGTATGCAGCATAGGCAGCGGCGGAGGCTGCTGCAGCCCCTGCTTTGG 161
GCKXPZK02GXA3T               315- AGACACATTGTACCCGTATGCAGCATAGGCAGCGGCGGAGGCTGCTGCAGCCCCTGCTTTGG 254
GCT3NVP02FHZUN               372- AGACACATTGTACCCGTATGCAGCATAGGCAGCGGCGGAGGCTGCTGCAGCCCCTGCTTTGG 311
F29OZW002G1Z6W               428+ AGACACATTGTACCCGTATGCAGCATAGGCAGCGGCGGAGGCTGCTGCAGCCCCTGCTTTGG 489
GCKXPZK01ANTDC               386+ AGACACATTGTACCCGTATGCAGCATAGGCAGCGGCGGAGGCTGCTGCAGCCCCTGCTTTGG 447
F7SNIN202F56NZ               118- AGACACATTGTACCCGTATGCAGCATAGGCAGCGGCGGAGGCTGCTGCAGCCCCTGCTTTGG 57
GCXRAE301CUGWE               138- AGACACATTGTACCCGTATGCAGCATAGGCAGCGGCGGAGGCTGCTGCAGCCCCTGCTTTGG 77
F29OZW001B1GED               179- AGACACATTGTACCCGTATGCAGCATAGGCAGCGGCGGAGGCTGCTGCAGCCCCTGCTTTGG 118
F29OZW001DZ4XQ               188- AGACACATTGTACCCGTATGCAGCATAGGCAGCGGCGGAGGCTGCTGCAGCCCCTGCTTTGG 127
F7SNIN202IU3H8               243- AGACACATTGTACCCGTATGCAGCATAGGCAGCGGCGGAGGCTGCTGCAGCCCCTGCTTTGG 182
GCQEMSA02GKPYC               213+ AGACACATTGTACCCGTATGCAGCATAGGCAGCGGCGGAGGCTGCTGCAGCCCCTGCTTTGG 274
GCT3NVP02GQKZI               168+ AGACACATTGTACCCGTATGCAGCATAGGCAGCGGCGGAGGCTGCTGCAGCCCCTGCTTTGG 229
F29OZW001AL3SG               208+ AGACACATTGTACCCGTATGCAGCATAGGCAGCGGCGGAGGCTGCTGCAGCCCCTGCTTTGG 269
                                                                *
```

B

```
Cfa1                    17040181+ GCAACGGCTTCCC-GTTCCGACAGAGAACCGTTTGGCACTTGCCCACGTTAGCAGAGGT 17040238
                                                               *
GCQEMSA02FW62Y                52+ GCAACGGCTTCCCCGTTCCGACAGAGAACCGTTTGGCACTTGCCCACGTTAGCAGAGGT 110
GCXRAE302JAGQY               209+ GCAACGGCTTCCC-GTTCCGACAGAGAACCGTTTGGCACTTGCCCACGTTAGCAGAGGT 266
GCT3NVP01DYBLQ               105- GCAACGGCTTCCC-GTTCCGACAGAGAACCGTTTGGCACTTGCCCACGTTAGCAGAGGT 48
F7SNIN201CYJG3               107- GCAACGGCTTCCC-GTTCCGACAGAGAACCGTTTGGCACTTGCCCACGTTAGCAGAGGT 50
F7SNIN201CJR21               124- GCAACGGCTTCCC-GTTCCGACAGAGAACCGTTTGGCACTTGCCCACGTTAGCAGAGGT 67
GCQEMSA02F6B97               155- GCAACGGCTTCCC-GTTCCGACAGAGAACCGTTTGGCACTTGCCCACGTTAGCAGAGGT 98
GCT3NVP02GSS4W               231- GCAACGGCTTCCC-GTTCCGACAGAGAACCGTTTGGCACTTGCCCACGTTAGCAGAGGT 174
GCXRAE301AVNEO               119+ GCAACGGCTTCCC-GTTCCGACAGAGAACCGTTTGGCACTTGCCCACGTTAGCAGAGGT 176
GCT3NVP01AM9J2               161- GCAACGGCTTCCC-GTTCCGACAGAGAACCGTTTGGCACTTGCCCACGTTAGCAGAGGT 104
GCKXPZK01BGUYI               415- GCAACGGCTTCCC-GTTCCGACAGAGAACCGTTTGGCACTTGCCCACGTTAGCAGAGGT 358
                                                               *
GCKXPZK02HKHIC                72- GCAACGGCTTCCC-GTTCCGACAGAGAACAGTTTGGCACTTGCCCACGTTAGCAGAGGT 15
F29OZW001D9D8R               172+ GCAACGGCTTCCC-GTTCCGACAGAGAACAGTTTGGCACTTGCCCACGTTAGCAGAGGT 229
F29OZW001EGPN4               160+ GCAACGGCTTCCC-GTTCCGACAGAGAACAGTTTGGCACTTGCCCACGTTAGCAGAGGT 217
GCKXPZK02HEX5B               176+ GCAACGGCTTCCC-GTTCCGACAGAGAACAGTTTGGCACTTGCCCACGTTAGCAGAGGT 233
GCXRAE302HN7NM               127+ GCAACGGCTTCCC-GTTCCGACAGAGAACAGTTTGGCACTTGCCCACGTTAGCAGAGGT 184
GCKXPZK01EM0HW               158- GCAACGGCTTCCC-GTTCCGACAGAGAACAGTTTGGCACTTGCCCACGTTAGCAGAGGT 101
GCXRAE302JP0IE               175+ GCAACGGCTTCCC-GTTCCGACAGAGAACAGTTTGGCACTTGCCCACGTTAGCAGAGGT 232
GCXRAE301EYRS3               302+ GCAACGGCTTCCC-GTTCCGACAGAGAACAGTTTGGCACTTGCCCACGTTAGCAGAGGT 359
F7SNIN201EHQQK                42+ GCAACGGCTTCCC-GTTCCGACAGAGAACAGTTTGGCACTTGCCCACGTTAGCAGAGGT 99
F29OZW001DWEKU               490- GCAACGGCTTCCC-GTTCCGACAGAGAACAGTTTGGCACTTGCC-ACGTTAGCAGAGGT 434
                                                               *
```
