# Supplementary material for: Sequence comparison of prefrontal cortical brain transcriptome from a tame and an aggressive silver fox (Vulpes vulpes)
Source: BMC Genomics. 2011 Oct 3;12:482. doi: 10.1186/1471-2164-12-482 (PMC3199282; doi:10.1186/1471-2164-12-482)
Supplement: Additional file 5 — Supplementary Table 2. Results of SNP validation by Sanger sequencing. Three sets (a, b, c) of SNPs were selected for validation by the following criteria: for set (a) the minor allele had to be present in at least 25% of the sequencing reads per sample and each SNP allele had to be represented in more then three reads per allele for an individual; for set (b) the minor allele had to be present in at least 25% of the sequencing reads per sample and each SNP allele in at exactly three reads per allele for an individual; and for set (c) the minor allele had to be present in at least three reads between the two individuals. Validation was undertaken by Sanger sequencing SNP-specific amplicons amplified from aliquots of the same cDNA submitted for 454 transcriptome sequencing. For each SNP the allele(s) identified in the tame and aggressive samples by 454 sequencing are tabulated with the number of 454 reads supporting the allele(s), together with the allele(s) identified by Sanger sequencing. For SNPs for which an allele was identified in a sample by one method (454 or Sanger) but not the other, the alleles are indicated in bold font, and if the allele was not found by 454 sequencing the number of reads is zero. [file 1471-2164-12-482-S5.PDF]

SNP set	SNP number	Tame			Aggressive		
		454	# reads	Sanger	454	# reads	Sanger
**a**	#1-1	G	36	G	A	32	A
	#1-2	A	37	A	G	25	G
	#1-3	**C/T**	**18/9**	**C**	**C/T**	**14/12**	**C**
	#1-4	T	32	T	C	21	C
	#1-5	C	16	C	T	18	T
	#1-6	G	11	G	C	17	C
	#1-7	G	9	G	A	15	A
	#1-8	G	7	G	A	12	A
	#1-9	T	6	T	C	13	C
	#1-10	G	10	G	A	8	A
	#1-11	G	6	G	A	10	A
	#1-12	T	7	T	C	9	C
	#1-13	C	7	C	G	8	G
	#1-14	A	5	A	G	10	G
	#1-15	T	8	T	C	5	C
	#1-16	T	8	T	C	4	C
	#1-17	C	7	C	T	5	T
	#1-18	T	7	T	C	5	C
	#1-19	A/G	4/4	A/G	G	5	G
	#1-20	A	5	A	**T**	**4**	**T/A**
	#1-21	C	4	C	**T**	**4**	**T/C**
**b**	#2-1	G	6	G	A/G	3/3	A/G
	#2-2	T	5	T	T/C	4/3	T/C
	#2-3	T	3	T	**C**	**5**	**C/T**
	#2-4	C	4	C	**T**	**3**	**T/C**
	#2-5	T	4	T	C	3	C
	#2-6	G	3	G	A/G	3/3	A/G
	#2-7	A	3	A	**G**	**3**	**G/A**
	#2-8	T	3	T	C	3	C
	#2-9	G	3	G	A	3	A
	#2-10	A	3	A	T	3	T
	#2-11	G	3	G	A	4	A
	#2-12	T	3	T	C	3	C
	#2-13	G	3	G	**A**	**3**	**A/G**
	#2-14	T	3	T	C	3	C
	#2-15	**A**	**3**	**A/G**	G	3	G
	#2-16	**-**	**0**	**T**	T/C	3/3	T/C
	#2-17	**-**	**0**	**T/C**	T/C	3/3	T/C
**c**	#3-1	G/A	3/1	G/A	**A**	**2**	**A/G**
	#3-2	G/A	2/2	G/A	G/A	1/1	G/A
	#3-3	G/A	2/2	G/A	A/G	2/1	A/G
	#3-4	C/T	2/1	C/T	T/C	2/1	T/C
	#3-5	C/T	2/1	C/T	T/C	2/1	T/C
	#3-6	**A/G**	**2/1**	**A**	G/A	2/1	G/A
	#3-7	C/T	2/1	C/T	T/C	2/1	T/C
	#3-8	A/G	2/2	A/G	A/G	1/1	A/G
	#3-9	T/C	1/1	T/C	T/C	2/2	T/C
	#3-10	T/G	2/1	T/G	G/T	2/1	G/T
	#3-11	A/G	1/1	A/G	A/G	2/2	A/G
	#3-12	T/C	2/2	T/C	T/C	1/1	T/C
	#3-13	C/T	2/1	C/T	T/C	2/1	T/C
	#3-14	T/C	1/1	T/C	T/C	2/2	T/C
	#3-15	**G/A**	**2/1**	**A**	**A/G**	**2/1**	**A**
