# Supplementary material for: Sequence comparison of prefrontal cortical brain transcriptome from a tame and an aggressive silver fox (Vulpes vulpes)
Source: BMC Genomics. 2011 Oct 3;12:482. doi: 10.1186/1471-2164-12-482 (PMC3199282; doi:10.1186/1471-2164-12-482)
Supplement: Additional file 8 — Supplementary Table 4. Primers for RT-qPCR experiments. Forward and reverse primers are listed that were used to amplify gene-specific fox amplicons by RT-qPCR to validate expression differences identified by transcriptome sequence analysis. [file 1471-2164-12-482-S8.PDF]

Primer name	Sequence	Amplicon size
HTR2CIF1	TGTTGAGCACAGCCGTTTC	145 bp
HTR2CIR1	GCACGTGGTGTTGTTGACG	
ITGA8IF1	ACTGAGGTCTTCGGGAGATTC	118 bp
ITGA8IR1	CTTGGCCTCTTTGATCCTTG	
LCORIF1	CATTCGCTGCATGATCTCAC	99 bp
LCORIR1	GCAGAAGAGTTCCTCAATGC	
CDONIF1	GCTGAACTGCGGAAACTCTC	108 bp
CDONIR1	CCCTCCTTCCAAACTTTCTG	
LRRC20IF1	TTGTTAGCCAGCGTGATGAG	143 bp
LRRC20IR1	GCAAGGAAGGTCAACGAGAC	
PRRG2IF1	AGGACAATGAGCAGGATGC	138 bp
PRRG2IR1	GCGGGAGTATTTCGAGGAC	
SLITRK6IF1	AACATCTCTTCCAGCTGTGC	97 bp
SLITRK6IR1	GAATCCACAGCTTCATGTTG	
SCGNIF1	GGGTTTCCTGACGAAAGAAC	134 bp
SCGNIR1	TGTCCAGAGGATGAAACAGC	
KCNMA1IF1	CAACAAGTACTGCGCTGACC	149 bp
KCNMA1IR1	TCCAGCTTGGGATGTTTAGC	
HPRT1F1	TGGAGATGACCTCTCAACTTTAAC	174 bp
HPRT1R2	GGGTTTATAGCCAACACTTCG	
